# Early preclinical development of *Mycobacteriumtuberculosis* amino acid biosynthesis pathway inhibitor DRILS-1398 as a potential anti-TB drug

**DOI:** 10.1016/j.isci.2025.112537

**Published:** 2025-04-29

**Authors:** Deepesh Biswas, Rebecca Kristina Edwin, K. Shiva Kumar, Anwar Alam, Dhiraj Kumar, Sandipan Chakraborty, Gopalakrishnan Bulusu, Farhan Jalees Ahmad, Gautham G. Shenoy, Lakshyaveer Singh, Mansi Agarwal, Fouzia Siraj, Srinivas Oruganti, Parimal Misra, Nasreen Zafar Ehtesham, Manojit Pal, Seyed Ehtesham Hasnain

**Affiliations:** 1 Dr. Reddy’s Institute of Life Sciences, University of Hyderabad Campus, Gachibowli, Hyderabad 500046, India; 2 Manipal College of Pharmaceutical Sciences, Manipal Academy of Higher Education, Madhav Nagar, Manipal 576104, India; 3 Department of Life Sciences, School of Basic Sciences and Research, Sharda University, Greater Noida 201310, India; 4 Department of Biotechnology, School of Engineering and Technology, Sharda University, Greater Noida 201310, India; 5 Cellular Immunology Group, International Centre for Genetic Engineering and Biotechnology, New Delhi 110067, India; 6 Department of Pharmaceutics, School of Pharmaceutical Education and Research, Jamia Hamdard, New Delhi 110062, India; 7 Tuberculosis Aerosol Challenge Facility, International Centre of Genetic Engineering and Biotechnology, New Delhi 110067, India; 8 Indian Council of Medical Research-Centre for Cancer Pathology, Safdarjung Hospital Campus, New Delhi 110029, India; 9 Department of Biochemical Engineering and Technology, Indian Institute of Technology, Hauz Khas, New Delhi 110016, India

**Keywords:** Biological sciences, Medical Microbiology, Microbiology, Natural sciences, Pharmacology

## Abstract

The search for new anti-tubercular agents is vital for the fight against *Mycobacterium tuberculosis*, particularly given the rise of drug-resistant strains. DRILS-1398, a pyrazolo[4,3-*d*]pyrimidine derivative, was discovered as a potent inhibitor of *M.tb* chorismate mutase (*M.tb*-CM) with an IC_50_ = 3.0 ± 0.2 *μ*M (*n* = 3) and IC_90_ = 10 *μ*M. The compound demonstrated efficacy against multi-drug resistant *M.tb* strains (MIC = 4 μg/mL, ∼10.0 μM) and effective inhibition of intracellular *M.tb* in THP-1 macrophages. With favorable pharmacokinetics, moderate stability *in vitro*, and a promising safety profile, DRILS-1398 showed no toxicity at doses up to 500 mg/kg b.w./day when dosed orally daily once for 7 consecutive days in mice. Both DRILS-1398 and its formulation DRILS-1398(F) were successful in clearing *M.tb* infection from the lungs and spleen in murine models. These findings suggest DRILS-1398 as a promising lead candidate for developing a first-in-class anti-tubercular drug.

## Highlights

• DRILS-1398 is a 1^st^ in class inhibitor of *M.tb* amino acid biosynthetic pathway
• DRILS-1398 is effective against intracellular MDR-*M.tb*
• Formulated DRILS-1398 elicits superior pharmacokinetics properties and efficacy
• DRILS-1398 is safe, non-toxic, and well-tolerated in animal models

## Graphical abstract

**Figure:**
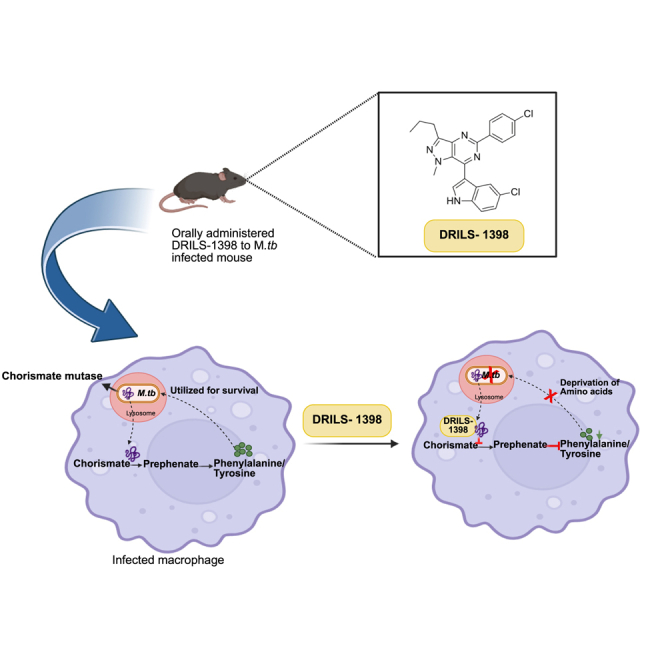

## Introduction

Tuberculosis (TB), caused by *Mycobacterium tuberculosis* (*M.tb*), is a global emergency accounting for 10.8 million cases per year, and the estimated TB death is 1.25 million in 2023, surpassing SARS-CoV 2 (WHO 2024, https://www.who.int/news-room/fact-sheets/detail/tuberculosis). *M.tb* has proven to be one of the most successful opportunistic pathogens and can undergo phenotypic adaptation during stress conditions to form biofilms^1^ that enable the bacteria to develop drug tolerance. *M.tb* possesses a wide repertoire of proteins that aid in establishing niche within the phagocytic-macrophage cells,^2^^,^^3^ suppressing phago-lysosomal fusion,^4^^,^^5^^,^^6^ evading immune cells,^7^^,^^8^ and modulating/counteracting the host immune responses.^9^^,^^10^^,^^11^ Several of these proteins play pivotal roles in the pathophysiology of *M.tb* and have been tested as drug targets.^1^^,^^12^^,^^13^ Management of TB has become very challenging^14^ due to the emergence of drug resistance, a subclinical condition where *M.tb* incorporates spontaneous resistance-causing mutations in drug target genes due to insufficient or incomplete anti-TB therapy.^15^^,^^16^

Focus on COVID-19 in the last couple of years has offset global efforts in the TB eradication program^10^^,^^17^ and has opened up new challenges for timely delivery of WHO (World Health Organization) End TB goals. With a ∼6% increase in TB incidence rate in 2021, compared to 2020, the nearly 2% annual decline seen over the past two decades has been reversed. To combat the exacerbated virulence and resistance pattern of the pathogen and to eliminate TB globally by 2035,^17^^,^^18^ it is critical to identify new drug targets as well as novel anti-TB drugs,^14^ including repurposed drugs.^19^ The current treatment for TB involves administering a combination of four drugs: isoniazid, rifampicin, pyrazinamide, and ethambutol.^20^ While this therapy has been effective in combating the disease thus far, its lengthy duration has prompted researchers to seek new inhibitors. Target-specific methods for identifying these inhibitors have gained traction due to their ability to allow medicinal chemists to adjust the pharmacodynamics and pharmacokinetics of drugs. This approach also enables them to optimize dosages and enhance the cellular uptake of molecules. The shikimic acid pathway, for instance, is exclusive to plants and microorganisms. This biosynthetic pathway is utilized to synthesize aromatic ring-structured compounds from carbohydrates and is essential for the survival of microorganisms. Chorismic acid lies at the terminal end of the shikimic acid pathway, where it is then converted to prephenate with the help of the enzyme chorismate mutase (CM), which ultimately leads to the synthesis of phenylalanine and tyrosine.^21^ Here, we describe the early preclinical development of a new anti-TB drug that inhibits *M.tb* CM,^22^ an enzyme absent in the human host. The *M.tb* CM gene, annotated as Rv1885c, is thus a potential therapeutic target.^23^

## Results

### Design and synthesis of DRILS-1398

**Figure 1:**
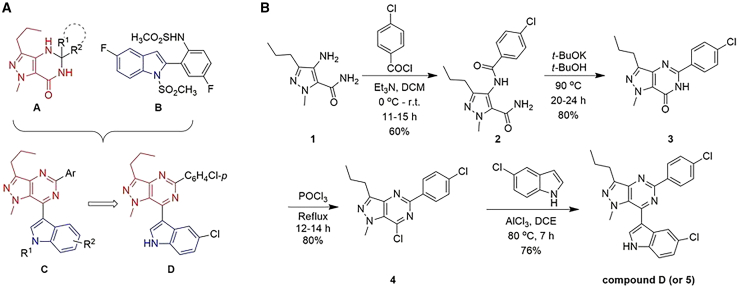
Design and synthesis of DRILS-1398 (A) Previously reported *M.tb*-CM inhibitors A and B, the newly designed framework C, and the identified new inhibitor DRILS-1398. (B) Synthesis of DRILS-1398.

*M.tb* CM is an attractive target for developing potential anti-TB drugs due to its exclusive presence in mycobacteria and absence in higher-order eukaryotes. Thus, efforts were devoted for developing small organic molecules as inhibitors of this enzyme, which was reviewed and reported in 2017.^23^ Indeed, a range of compounds containing carbocyclic moiety initially and then aromatic ring, and subsequently the heterocyclic structure were reported to be inhibitors of *M.tb*-CM *in vitro*. The elegant synthesis of transition state analogs way back in 1988,^24^ the initial report on the identification of arene-based molecules by using a combination of ligand-based virtual screening and biological assays in 2007,^25^ the report on the first example of *M.tb*-CM inhibition by a heteroarene-based small molecule in 2011^26^ etc. were the key progresses observed during this period. While some showed moderate to good inhibition of this enzyme as reflected by their IC_50_ values, further clinical development of those molecules were not reported. Since 2017, continued research efforts from our group led to the identification of small organic molecule-based inhibitors,^27^^,^^28^^,^^29^^,^^30^ some of them interestingly showed effects on *M.tb* cell viability. The heteroarenes explored in these studies are (pyrazolo/benzo) triazinones, isatin-indole derivatives, benzofuran-2-ylmethyl derivatives, pyrazolo [4,3-*d*] pyrimidinones etc. Nevertheless, in some of our previous studies, the fragment 4-amino-1-methyl-3-propyl-1-*H*-pyrazole-5-carboxamide was found in several inhibitors that showed activity against CM.^27^^,^^29^^,^^30^^,^^31^ One such class of compounds is **A** (Figure 1A). Two hit compounds based on **A** decreased the viability of *M.tb in vitro* (IC_50_ ∼10–30 μM).^30^ On the other hand, an indole derivative **B** (Figure 1A) showed moderate inhibition of *M.tb*-CM (IC_50_ ∼17.02 μM).^26^ We used scaffold-merging techniques to design the new framework **C** (Figure 1A) that was used to create a library of small molecules for pharmacological studies. Thus, synthesis and subsequent *in vitro* evaluation of these molecules led to the identification of DRILS-1398 as a promising hit (World Patent Application WO 2020/240272 A1). Notably, we have tried to develop the structure-activity relationship (SAR) based on analogs of DRILS-1398. During the *in vitro* activity evaluation of these compounds against *M.tb*-CM, the fine precipitation of compounds in the assay medium was observed, which posed a challenge in determining the activities of several compounds. DRILS-1398 was synthesized via a multi-step reaction sequence (Figure 1B) that involved the amidation of amine **1** to afford the amide **2**, which on *t*-BuOK-mediated intramolecular cyclization produced the compound **3**. On chlorination using POCl_3,_ compound **3** afforded the chloro derivative **4**, which on AlCl_3_ induced heteroarylation with 5-chloroindole, resulted in DRILS-1398 (White solid; mp 230°C–232°C; ^1^H NMR [400 MHz, CDCl_3_] δ 8.79 [bs, 1H, NH], 8.54 [d, *J* = 9.2 Hz, 2H, ArH], 8.04 [d, *J* = 2.0 Hz, 1H, ArH], 7.68 [d, *J* = 3.2 Hz,1H, ArH], 7.47–7.40 [m, 3H, ArH], 7.31–7.25 [m, 1H, ArH], 3.94 [s, 3H, NCH_3_], 3.12 [t, *J* = 7.6 Hz, 2H, CH_2_], 2.04–1.97 [m, 2H, CH_2_], 1.10 [t, *J* = 7.4 Hz, 3H, CH_3_]; ^13^C NMR [100 MHz, CDCl_3_] δ 156.2, 147.1, 146.5, 145.7, 136.9, 135.7, 134.5 [2C], 129.3 [2C], 128.6 [2C], 127.9, 127.6, 127.5, 124.0, 120.3, 113.2, 112.6, 38.9 [NMe], 29.6 [CH_2_], 22.1 [CH_2_], 14.1 [Me]; IR [KBr] v_max_3206, 3170, 2957, 1541, 1109 cm^−1^; HRMS [ESI] calculated for C_23_H_20_N_5_Cl_2_ [M + H]^+^ 436.1109, found 436.1096; HPLC: 98.57%; for further details, see Figures S1–S5 in the supplemental information). Envisioning the medicinal significance of DRILS-1398 as a potential anti-TB drug, further *in vitro* and *in vivo* studies were pursued.

### DRILS-1398 is a potent inhibitor of *M.tb*-CM

**Figure 2:**
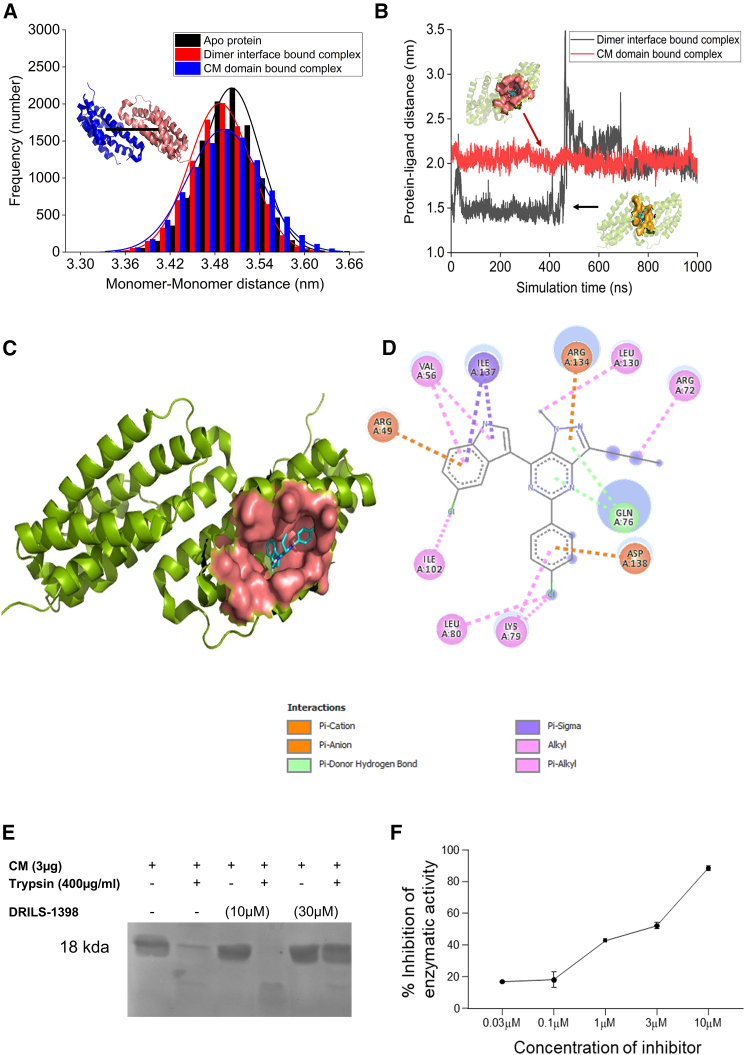
DRILS-1398 binds to the chorismate binding domain of *M.tb*-CM and inhibits the enzyme activity in a dose-dependent manner (A) Center of mass distance distributions between two monomers of chorismate mutase obtained from 1 μs simulation trajectories of apo-protein and bound to DRILS-1398 at two different sites. (B) Changes in the center of mass distances between ligand and protein dimers with simulation time for compound bound to two different binding sites of the protein. (C) Representation of stable DRILS-1398-CM bound complex. The protein is depicted as a green ribbon, and the binding site is shown in a pink surface representation. The docked conformation of DRILS-1398 is shown as a cyan stick. (D) Pharmacophoric features for the interaction of DRILS-1398 to site 1 of the *M.tb*-CM. (E) Protease protection assays of *M.tb*-CM protein in the presence of different concentrations of DRILS-1398. (F) Concentration-response curve for the inhibition of the enzymatic activity of the recombinant *M.tb*-CM. Each data point represents the mean of three independent experiments and its standard deviation.

DRILS-1398 was designed based on a CM inhibitory scaffold. We have used molecular docking and extensive all-atom molecular dynamic simulations to check the binding details and inhibition mechanism of DRILS-1398 on *M.tb*-CM. The crystal structure of the secretory *M.tb*-CM dimer (PDB ID: 2F6L)^32^ was probed for possible ligand binding pocket identification using PrankWeb.^33^ There are three potential ligand-binding sites (Figure S6): one subunit possesses a comparatively larger binding pocket (site 1) than the pocket in the other subunit (site 2), as the dimer is asymmetric. The third binding pocket is at the interface between two dimers (site 3). Molecular docking of DRILS-1398 to individual binding pockets revealed that the compound preferably docked to site 1 and site 3 with comparable affinity (Figure S6). However, the binding affinity of DRILS-1398 was significantly lower when bound to the shallow binding site 2. We further used extensive molecular dynamics simulations to delineate the interaction details of DRILS-1398 with *M.tb*-CM. Notably, the binding of DRILS-1398 at the CM domain (site 1) or the interface region of the dimer (site 3) does not dissociate the CM dimer, as the center of mass distance distributions obtained from simulation trajectories remained unaltered in comparison to the apo dimer complexes (Figure 2A). Further analysis revealed that DRILS-1398 docked strongly to the CM domain (site 1) and maintained the bound conformation throughout the 1 μs simulation timescale (red line, Figure 2B). However, the interfacial docked conformation of the compound was not stable during the simulation. The compound dissociated from the binding site ∼100 ns and exhibited non-specific binding at the interfacial region up to 400 ns. A complete dissociation of the ligand from the dimer interface and rebinding to site 1 of the CM domain was observed. We noted reorganization of the surrounding helices within 500–650 ns timescale to appropriately accommodate the ligand into the binding site, which remained stable during the remaining simulation timescale (Figure 2B). Our simulation data suggest that DRILS-1398 is capable of binding to the active site of the *M.tb*-CM and form a stable complex (Figure 2C). We explored the pharmacophoric features of interactions for DRILS-1398 bound to site 1 (Figure 2D). Further analysis of the stability of the dominant interactions throughout the 1 μs simulation trajectory revealed that the formation of specific H-bonds, π-cation, π-anion, and π-alkyl interactions within the catalytic site of *M.tb*-CM is the driving force for effective ligand recognition (Figure S7). The GLN76 formed a stable H-bond with the compound. The ARG49 and ARG134 formed very stable π-cation interactions with DRILS-1398, while the π-anionic interaction with ASP138 was only transiently stable. The ILE137 formed stable π-alkyl interactions with the compound (Figure S7).

We have further performed *in vitro* bio-assays to substantiate the binding and inhibition mechanism of *M.tb*-CM by DRILS-1398, as proposed from the molecular dynamics simulation. Protease-protection assay^22^ further confirmed the binding of DRILS-1398 to *M.tb*-CM, as 30 *μ*M of DRILS-1398 was resistant to trypsin digestion. It was hypothesized that the binding of DRILS-1398 caused conformational changes of *M.tb-*CM and became resistant to proteolytic cleavage (Figure 2E). DRILS-1398 emerged as a hit molecule by evaluating a series of compounds based on framework **C** (Figure 1A) against *M.tb*-CM *in vitro* using an enzymatic bio-assay. In the *in vitro* substrate competition assay,^32^ DRILS-1398 showed concentration-dependent inhibition of *M.tb*-CM activity with an IC_50_ = 3.0 ± 0.2 *μ*M (*n* = 3) and IC_90_ = 10 *μ*M (Figure 2F). Direct inhibition of the catalytic function of *M.tb*-CM by DRILS-1398 indicates its binding to the catalytic domain of the protein, reinforcing our prediction from molecular dynamics simulations.

### *In vitro* amino acid supplementation experiments demonstrate that DRILS-1398 inhibits *M.tb* CM

**Figure 3:**
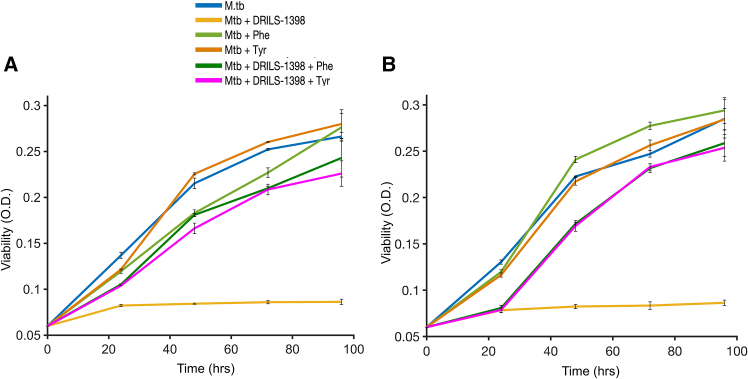
*In vitro* amino acid supplementation experiments demonstrate that DRILS-1398 inhibits *M.tb* CM (A) *M.tb* H_37_Rv cells (OD_600_ 0.06) were cultured in the presence of amino acids and DRILS-1398 at time = 0. The growth of bacilli was monitored at 24 h, 48 h, 72 h, and 96 h. *M.tb* H_37_Rv cells untreated with amino acid and DRILS1398 were taken as control. (B**)***M.tb* H_37_R_v_ cells (OD_600_ 0.06) were cultured. At time = 0, DRILS-1398 was added. After 24 h, amino acids were added to the culture, and the growth of bacilli was monitored at 24 h, 48 h, 72 h, and 96 h. *M.tb* H_37_Rv cells untreated with amino acid and DRILS1398 were taken as control.

In order to assess the role of DRILS-1398 in inhibiting *M.tb* CM, we performed rescue experiments using amino acids tyrosine and phenylalanine. *M.tb* H_37_Rv cells were taken in the log phase and treated with DRILS-1398 (∼10 μM) and in combination with tyrosine (100 μg/mL) or phenylalanine (100 μg/mL) at time = 0. Cell growth was assessed at time 24 h, 48 h, 72 h, and 96 h spectrophotometrically. Appropriate controls are also considered (Figure 3A). It was observed that amino acid supplementation rescued the cells from the inhibitory effects of DRILS-1398 (Figure 3A). Alternatively, *M.tb* H_37_Rv cells were taken in the log phase and treated with DRILS-1398 (4 μg/mL). After 24 h, cells were treated with tyrosine (100 μg/mL) or phenylalanine (100 μg/mL) and incubated until 96 h. DRILS-1398 showed inhibitory effects up to 24 h. When the amino acids were added to the culture at time = 24 h, it significantly restored cell growth (Figure 3B). DRILS- 1398 treatment led to suppression of amino acids of the CM pathway. In the absence of the amino acid, the bacteria failed to grow. However, the bacilli could resume growth when the culture was supplemented with amino acids. This observation further reinforces that DRILS-1398 is possibly targeting a crucial enzyme involved in the amino acid biosynthesis pathway, and our *in vitro* enzymatic assay and detailed atomistic simulation suggest CM is possibly the target.

### Efficacy of DRILS-1398 against MDR-*M.tb* ATCC-35825 and intracellular *M.tb* ATCC-27294, ATCC-35825 in activated THP-1 macrophages

**Figure 4:**
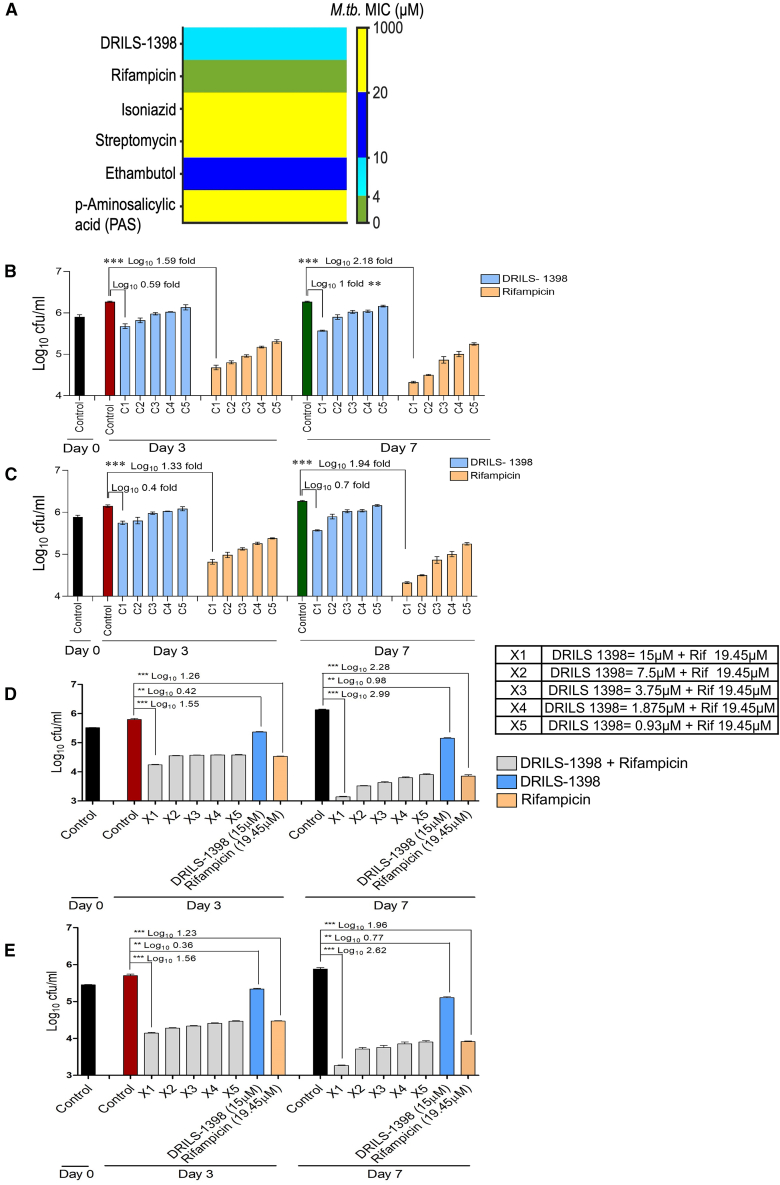
Efficacy of DRILS-1398 against MDR-*M.tb* ATCC-35825 and intracellular *M.tb* ATCC-27294, ATCC-35825 in activated THP-1 macrophages (A) Broth dilution MIC of DRILS-1398 and other marketed drugs against MDR-*M.tb* ATCC-35825. (B) Comparison of the log_10_ cfu/mL reduction ability of DRILS-1398 and rifampicin in different time points (day 0, day 3, and day 7) in intracellular efficacy assay with *M.tb* ATCC-27294 in THP-1 macrophages. (C) Comparison of the log_10_ cfu/mL reduction ability of DRILS-1398 and rifampicin in different time points (day 0, day 3, and day 7) in intracellular efficacy assay with MDR-*M.tb* ATCC-35825 in THP-1 Macrophages. DRILS-1398 was tested at C1:15 μM, C2:10μM, C3: 3μM, C4: 1 μM, C5: 0.3 μM. Rifampicin was tested at C1: 13.16 μM, C2: 6.58 μM, C3: 3.29 μM, C4: 1.65 μM, C5: 0.82μM. (D) Comparison of the log_10_ cfu/mL reduction ability of DRILS-1398 in combination with rifampicin on different days (day 0, day 3, and day 7) in intracellular efficacy assay with *M.tb* strain (ATCC-27294) in THP-1 macrophages. (E) Comparison of the log_10_ cfu/mL reduction ability of DRILS-1398 in combination with rifampicin in different time points (day 0, day 3, and day 7) in intracellular efficacy assay with MDR-*M.tb* strain (ATCC-35825) in THP-1 macrophages. X1: DRILS-1398 15μM + Rif 19.45 μM, X2: DRILS-1398 7.5μM + Rif 19.45 μM, X3: DRILS-1398 3.75μM + Rif 19.45 μM, X4: DRILS-1398 1.875μM + Rif 19.45 μM, X5: DRILS-1398 0.93μM + Rif 19.45 μM. Values are shown as mean ± SEM; ∗∗*p* < 0.01, ∗∗∗*p* < 0.001, ∗∗∗∗*p* < 0.0001 versus corresponding control using One-way ANOVA followed by Dunnetts multiple comparison test.

DRILS-1398 showed promising inhibitory efficacy against the multi-drug resistant *M.tb* strain (*Mycobacterium tuberculosis* ATCC-35825). The MIC (minimum inhibitory concentration) of DRILS-1398 was 4 μg/mL (∼10.0 μM) against MDR-*M.tb* ATCC 35825 (Figure 4A). The MIC of DRILS-1398 against the *M.tb.* ATCC-27294 strain was 6.525 μg/mL (∼15 μM). The efficacy of DRILS-1398 was then evaluated against *M.tb* in the monocytic macrophage cell line THP-1.^34^^,^^35^ After 72 h of activation, the THP-1 macrophages were infected with *M.tb-*H_37_R_v_ strain ATCC 27294 and multi-drug resistant strain ATCC 35825, at a multiplicity of infection (MOI) of 1:10. The *M.tb*-infected macrophages were exposed to DRILS-1398 or the standard drug, rifampicin. At the highest concentration tested (15.0 μM), DRILS-1398 showed a reduction of 0.59 log_10_ cfu/mL on day 3 compared to day-3 control (Figure 4B) in the intracellular efficacy THP-1 macrophage assay against *M.tb* H_37_Rv (ATCC 27294). Compared to day-7 control, cfu reduction was 1.00 log_10_ cfu/mL (Figure 4B). On the other hand, DRILS-1398 (15.0 μM) showed a reduction of 0.40 log_10_ cfu/mL on day 3 vs. day-3 control (Figure 4C), in the intracellular efficacy THP-1 macrophage assay against MDR-*M.tb* H_37_Rv (ATCC 35825). Compared to day-7 control, cfu reduction was 0.70 log_10_ cfu/mL (Figure 4C).

*M.tb*-infected macrophages were also exposed to DRILS-1398 in the presence of fixed concentration (19.45 μM) of rifampicin and varying concentrations of DRILS-1398 (15.0 μM, 7.5 μM, 3.75 μM, 1.875 μM, and 0.93 μM) respectively. In the intracellular efficacy THP-1 macrophage assay against *M.tb* H_37_R_v_ (ATCC 27294), on day 3, DRILS-1398 (15.0 μM) in combination with rifampicin at 19.45 μM showed a cfu reduction of 1.55 log_10_ cfu/mL, compared to day 3 control, whereas, rifampicin and DRILS-1398 alone showed a reduction of 1.26 log_10_ cfu/mL and 0.42 log_10_ cfu/mL alone, respectively, at this concentration. On day 7, the same combination dose demonstrated a reduction of 2.99 log_10_ cfu/mL, compared to the day 7 control (Figure 4D). Whereas rifampicin and DRILS-1398 alone showed a reduction of 2.28 log_10_ cfu/mL and 0.98 log_10_ cfu/mL alone, respectively, at this concentration showing an additive effect in this combination dose. While, in MDR-H_37_R_v_ (ATCC 35825) DRILS-1398 (15 μM) in combination with rifampicin (19.45 μM) showed a cfu reduction of 1.56 log_10_ cfu/mL on day 3 vs. day 3 control. On day 7, it demonstrated a decrease of 2.62 log_10_ cfu/mL compared to the day 7 control (Figure 4E). The combination of the highest dose of DRILS-1398 at 15 μM with 19.45 μM rifampicin showed superior cfu (2.62 log_10_ cfu/mL) clearance compared to individual compounds, DRILS-1398 (0.77 log_10_ cfu/mL) and rifampicin (1.96 log_10_ cfu/mL) at their highest doses, demonstrating additive effect in this combination dose. A detailed analysis of log_10_ cfu reduction and its statistical significance for each combination dosage against *M.tb-*H_37_R_v_ strain ATCC 27294 and a multi-drug resistant strain ATCC 35825 are shown in supplementary figures, Figures S8 and S9, respectively.

### Safety profiling of DRILS-1398

Safety profile of DRILS-1398 was evaluated to decide the tentative doses of the molecule for *in vivo* efficacy study. DRILS-1398 (3 μM) was incubated with pooled liver microsomes at 5 time points over 120 min and analyzed by LC-MS/MS. Mouse microsomal stabilities of the compound at different time points were 56.45% (2 min), 53.42% (5 min), 42.18% (15 min), 34.26% (30 min), 24.61% (45 min), and 26.57% (2 h) (Figure S10A). Results showed that the compound was moderately stable (∼35% at 30 min). Analysis of the percent of the unbound compound showed that DRILS-1398 was highly bound to the plasma protein (∼98.16%) (Figure S10B). DRILS-1398 did not show any mutagenic potential when tested with *Salmonella typhimurium* TA98, TA100, and TA1537 at 3000.0, 949.5, 300.0, 94.9, and 30.0 μg/plate concentrations with and without rat liver S9. Cytochrome P450 enzyme inhibition studies against major 5 CYP enzymes were performed for DRILS-1398 at 30 μM using Vivid CYP P450 blue screening kits by fluorescent-based method. The IC_50_ values of DRILS-1398 were >30, 12.22, 13.71, >30, and >30 μM against CYP 1A2, 2C9, 2C19, 2D6, and 3A4 enzymes, respectively (Table S1, Figure S11). DRILS-1398 showed CYP inhibition within the acceptable range. Blockade of the IKr potassium current constitutes an index of QT interval prolongation for cardiac risk assessment. The inhibitory effect of DRILS-1398 on the rapid component of the delayed rectifier potassium current (IKr) was investigated in HEK (human embryonic kidney) cells stably transfected with the hERG gene, employing the whole cell patch-clamp technique. DRILS-1398 at all concentrations 0.1, 0.3, 1, 3, 10, and 30 μM inhibited hERG current by 18.59% (±2.03), 32.32% (±5.14), 32.98% (±3.76), 39.09% (±3.29), 47.89% (±7.72), and 60.18% (±18.26) respectively, versus vehicle control current. Under similar conditions, the positive control (10 μM propafenone hydrochloride) inhibited hERG current by 95.85% (±1.86). The IC_50_ of DRILS-1398 is 10.18 μM. Thus, DRILS-1398 is a moderate inhibitor of hERG (IKr) current (Figure S12).

### Formulation of DRILS-1398 improves the pharmacokinetic and efficacy profile in the animal model

**Figure 5:**
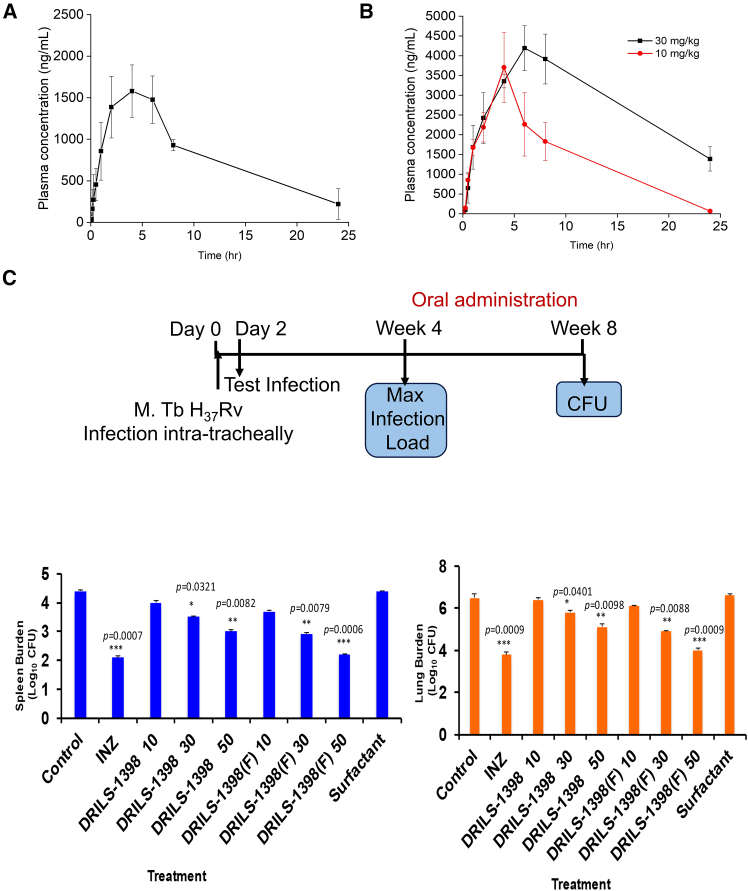
Formulated DRILS-1398 (DRILS-1398(F)) shows a better pharmacokinetic profile and *in vivo* efficacy (A) Single dose (50 mg/kg) oral pharmacokinetics study of DRILS-1398 in male BALB/c mice. (B) Oral pharmacokinetics study of formulated DRILS-1398(F) in mice at 10 and 30 mg/kg. Pharmacokinetics data were analyzed using Phoenix WinNonlin 8.1 software, and a non-compartmental model was selected for analysis. The mean and SD for the plasma concentrations were calculated using Microsoft Excel. (C) DRILS-1398 and DRILS-1398(F) were administered to mice infected with *M.tb* H_37_Rv, as mentioned in methodology. Treatment with isoniazid (INZ) was taken as control. Bacterial burden in the lung and spleen of drug-treated mice was assessed by CFU assay. The CFU from two duplicate agar plates was observed and log-transformed before analysis. Statistical significance was determined by two-way ANOVA. *p* < 0.05 was considered significant, ∗*p* < 0.05, ∗∗*p* < 0.01, ∗∗∗*p* < 0.001.

The *in vitro* safety profile of DRILS-1398 prompted us to conduct the oral and intravenous (IV) pharmacokinetics (PK) of the molecule. Male BALB/c mice were administered with DRILS-1398 at a dose of 50 mg/kg body weight orally using the formulation vehicle 1% (v/v) Tween 80 + 99% (v/v) of 0.5% (w/v) Carboxymethylcellulose sodium salt in ultrapure water. Animals did not show any adverse clinical signs during the treatment. The maximum plasma concentration (C_max_) of DRILS-1398 following oral administration was 1580.00 ng/mL. Plasma exposure (AUC last) of DRILS-1398 was 19189.66 h∗ng/mL, and the elimination half-life for the compound was 6.95 h (Figure 5A, Table S2). Single dose IV (10 mg/kg) pharmacokinetics study of DRILS-1398 in male BALB/c mice showed that the molecule stays in the circulation for a longer time (AUC = 4700 h∗ng/mL) and t_1/2_ was ∼8.25 h (Figure S13). The approximate free drug concentration in mice blood plasma at 10 mg/kg in the IV route was ∼0.4 μM. Considering the moderate stability of the molecule in mice plasma (∼35% in 30 min) and having a large clearance volume (2100 mL/kg/h), the free drug concentration should be > ∼0.4 μM. As the molecule is not water-soluble, the molecule could be dosed at a much higher concentration so that a higher free drug concentration could be achieved *in vivo*.

To improve the pharmacokinetic profile (PK) a formulated DRILS-1398 i.e., DRILS-1398(F) was prepared using micelles containing 0.3% drug, 20% Smix containing Tween 80: PEG 400 (1:1), and 79.7% normal saline. Male BALB/c mice were orally administered 10 mg/kg and 30 mg/kg of DRILS-1398(F). Blood samples were collected at specified time points and were analyzed for DRILS-1398 using an optimized LC-MS/MS method. PK studies of DRILS-1398(F) in mice at 10 mg/kg and 30 mg/kg showed ∼33 μg/mL and ∼66 μg/mL of the compound in the blood plasma level (Figure 5B, Table S3). The IC_50_ of the molecule was ∼3.0 μM, and the blood level of the formulated compound (Mol. wt. of DRILS-1398 is 430) was ∼153.0 μM at 30 mg/kg and ∼75.0 μM at 10 mg/kg, respectively. The levels were much higher than the IC_50_ value of the molecule.

After obtaining the oral PK data of both the non-formulated and formulated DRILS-1398, we assessed the efficacy of DRILS-1398 against *M.tb* infection *in vivo*. BALB/c mice (20–25g) were infected with *M.tb* H_37_Rv, and clearance of infection was assessed in the lungs and spleen. After four weeks of infection, mice (*n* = 6) were sacrificed, and lungs and spleen were excised, homogenized, and plated on 7H11-OADC agar plates. Mice showed distinct granuloma in the lungs, which was distinctive of *M.tb* infection load. After the fourth week of infection, different groups of control and *M.tb* H_37_Rv infected mice were administered the non-formulated DRILS-1398 and formulated DRILS-1398(F) drugs on alternate days. The following control and treatment groups of mice were considered: group 1: uninfected; group 2: infected with *M.tb* H_37_Rv; group 3: infected with *M.tb* H_37_Rv and treated with DRILS-1398 (10–50 mg/kg); group 4: Infected with *M.tb* H_37_Rv and treated with DRILS-1398(F) (10–50 mg/kg); group 5: infected with *M.tb* H_37_Rv and treated with isoniazid (5 mg/kg); group 6: Infected with *M.tb* H_37_Rv and treated with surfactant (Tween + PEG). After four weeks of administration of DRILS-1398 and DRILS-1398(F), the *M.tb* H_37_Rv infection was effectively cleared in the lungs and spleen, respectively (Figure 5C).

### Histopathology analysis

**Figure 6:**
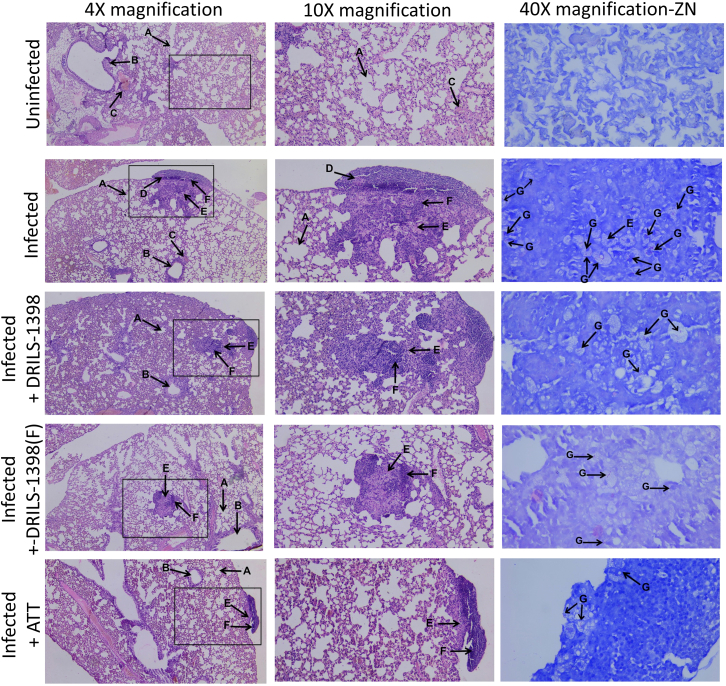
Histopathological analysis of the effect of DRILS-1398 and DRILS-1398(F) in clearance of infection BALB/c mice were infected with M.tb H_37_Rv. Lung sample from the uninfected, infected, infected + DRILS-1398 treatment, infected + DRILS-1398(F) and infected + ATT (Anti-Tuberculosis Therapy i.e. Isoniazid) mice were obtained, fixed and H&E staining was done to assess the presence of lymphocytes, histiocytes, and granuloma (horizontal panels). Histopathological analysis of the lungs is shown above at 4× magnification (Left vertical panel) and the inset depicted in 4x is shown at 10× magnification (middle vertical panel). The tissue sample section was stained with Ziehl-Neelsen (ZN) stain and the presence of acid-fast bacilli in the specimen was observed under 40× magnification (Right vertical panel). A-alveoli, B-bronchiole, C- blood vessel, D- granuloma, E- histiocytes, F-lymphocytes, G- bacilli.

Histopathological analysis of the lungs of the control and infected mice was carried out to assess the presence of granuloma and histio-lymphocytic cells. Uninfected mice (control) did not exhibit necrosis, granuloma, and histio-lymphocytic cells. The *M.tb*-H_37_Rv infected mice showed the presence of granuloma in the lung. The presence of lymphocytic cells in the lung tissue of the *M.tb*-H_37_Rv infected mice was also observed. Infected mice treated with drugs: DRILS-1398 (30 mg/kg), DRILS-1398(F) (30 mg/kg) and isoniazid (5 mg/kg), showed no signs of granuloma, indicating clearance of infection in the lung (Figure 6). ZN staining of lung specimens of infected mice was done to assess the presence of acid-fast bacilli post-drug treatment. It was observed that the isoniazid-treated group showed minimal presence of bacilli. Treatment of DRILS-1398 or DRILS-1398(F) also led to a significant decrease in bacilli load as compared to the infected group of mice, which were not provided any drugs (Figure 6, Table S4).

### Toxicity profile of DRILS-1398 in BALB/c mice

Having established the efficacy of DRILS-1398 against *M.tb* infection *in vivo*, it was necessary to assess its toxicity in an appropriate animal model. Forty males and forty female mice were assigned into four groups of ten animals/sex per group viz., vehicle control (G1), low dose (G2), mid dose (G3), and high dose (G4). The test item formulations were freshly prepared as a vehicle in ultra-pure water. The vehicle alone was administered to the vehicle control (G1) animals. Test item formulations were administered daily once at dose levels of 125 (G2), 250 (G3), and 500 (G4) mg/kg b.w./day. Repeated dose administration of DRILS-1398 up to 500 mg/kg b.w./day daily once for 7 consecutive days in BALB/c mice of both genders showed no adverse clinical signs of toxicity and mortality/morbidity throughout the experimental period. No treatment-related adverse effects were observed on body weight, weight gain, feed consumption, hematological, and clinical chemistry parameters. Furthermore, histopathological evaluation of the examined tissues revealed no treatment-related adverse effects of DRILS-1398 when administered by oral gavage daily once for 7 consecutive days (supplemental information, Figure S14).

## Discussion

There is an urgent requirement for the development of better treatments for MDR-TB. The first 6-month regimen was approved in 2019 for treating MDR and XDR with novel mechanisms of action. Later, the WHO consolidated guidelines on tuberculosis treatment, including two new recommendations for using a 4-month regimen composed of rifapentine, isoniazid, pyrazinamide, and moxifloxacin. However, a shorter, better-tolerated, and more efficacious therapy across all patient populations remains an unmet medical need. To achieve this goal, the incorporation of new therapeutic targets and novel drug combinations is highly essential.

Here, we report the development of DRILS-1398, a first-in-class small molecule inhibitor against *M.tb* CM. CM, an essential enzyme at the branch point of the shikimate pathway, is a unique and promising target for developing inhibitors with potential antimycobacterial activity. DRILS-1398 binds to CM and inhibits its activity at an IC_50_ of 3.0 ± 0.2 *μ*M and IC_90_ of 10 *μ*M. *In vitro* binding studies using protease-protection assays revealed that binding of DRILS-1398 to *M.tb*-CM caused conformational changes of *M.tb*-CM, rendering its resistant to proteolytic cleavage. Detailed characterization of the binding mechanism of DRILS-1398 was explored using molecular modeling, docking, and extensive molecular dynamics simulation studies. Molecular dynamics simulation convincingly demonstrated that DRILS-1398 binds to the *M.tb*-CM catalytic domain and inhibits the activity of the enzyme, which is evident from the *in vitro* enzymatic assay. Formation of specific H-bonds, π-cation, π-anion, and π-alkyl interactions within the catalytic site of *M.tb*-CM is the driving force for effective ligand recognition. Amino acid supplementation experiments further confirm the efficacy of DRILS-1398 as an inhibitor of the aromatic amino acid biosynthetic pathway of *M.tb* (where CM plays a critical role). We evaluated the effect of supplementation of the aromatic amino acids tyrosine and phenylalanine, which could rescue the growth of *M.tb* that was inhibited by treatment with DRILS-1398. It was observed that treating DRILS-1398 to H_37_Rv cells led to cell growth inhibition. Interestingly, supplementation of amino acids restored cell growth. This observation further reinforces that DRILS-1398 is possibly targeting a crucial enzyme involved in the amino acid biosynthesis pathway, and our *in vitro* enzymatic assay and detailed atomistic simulation suggest CM is possibly the target.

DRILS-1398 is efficacious against with *M.tb-*H_37_R_v_ strain ATCC 27294 and a multi-drug resistant strain ATCC 35825 in activated THP-1 macrophages. Furthermore, the combination of the highest dose of rifampicin at 19.45 μM and DRILS-1398 at 15.0 μM showed superior cfu clearance compared to individual compounds, DRILS-1398 and rifampicin, at their highest doses. Since DRILS-1398 showed potent activity against *M.tb*, we then evaluated the safety profile of the compound. Mouse microsomal stabilities showed that the compound was stable and highly protein-bound. DRILS-1398 was also non-mutagenic in the Ames test and showed an acceptable range of CYP inhibition ability. We then conducted the oral and intravenous pharmacokinetic studies in mice. The oral administration of DRILS-1398 at a dose of 50 mg/kg showed moderate PK, while administration of DRILS-1398 at 10 mg/kg (single dose) through the intravenous route improved the PK, with a longer half-life of ∼8.5 h and free drug concentration of 0.4 μM. To increase the free drug concentration and enhance the pharmacokinetic profile of DRILS-1398, we administered it in a formulated form, e.g., DRILS-1398(F), via the oral route at 10 and 30 mg/kg. DRILS-1398(F) in mice showed a superior PK profile both at 10 and 30 mg/kg dosages. Notably, the IC_50_ of the molecule was ∼3.0 μM, whereas the blood level of the formulated compound was ∼75.0 and ∼153.0 μM at 10 and 30 mg/kg, respectively. We further evaluated the *in vivo* efficacy of formulated DRILS-1398(F) and non-formulated DRILS-1398 in BALB/c mice infected with *M.tb* H_37_R_v_. After four weeks of administration, both DRILS-1398 and DRILS-1398(F) effectively cleared *M.tb* H_37_Rv infection in the lungs and spleen, respectively. DRILS-1398(F) showed enhanced bioavailability in mice and exhibited high efficiency in the *in vivo* mouse model. DRILS-1398 emerged as the first inhibitor of *M.tb*-CM that showed efficacy in an *in vivo* model.

Histopathological analysis of the lungs of the mice infected with *M.tb*-H_37_Rv showed the presence of granuloma, indicative of TB infection. The presence of lymphocytes in the lungs of the infected mice is indicative of the host immune response. Treatment of DRILS-1398 or DRILS-1398(F) led to clearance of infection, which was evident by the absence of granuloma. The isoniazid-treated infected mice also showed no signs of granuloma. The presence of acid-fast bacilli within the lung of infected mice was significantly reduced upon treatment with DRILS-1398 or DRILS-1398(F), which is comparable with isoniazid treatment. Necrosis of the lung tissue by treatment of DRILS-1398 or DRILS-1398(F) was not observed, indicating the drug dosing was safe.

**Scheme 1:**
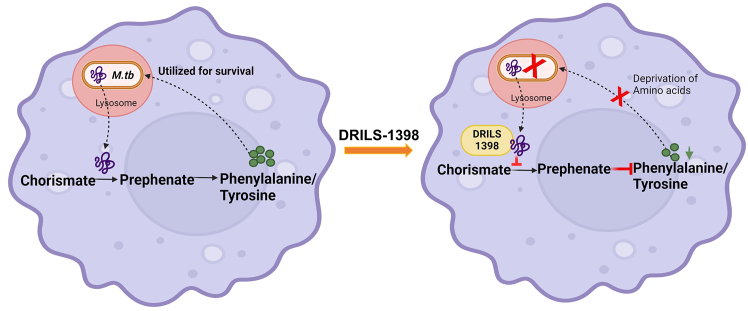
Probable mechanism of action of DRILS-1398 in inhibition of *M.tb* Left: Chorismate mutase present in the periplasmic space enables conversion of chorismate to prephenate which finally results in the production of amino acids Phenylalanine and Tyrosine, that are utilized for survival of the *M.tb* bacterium within the macrophage. Right: DRILS-1398 binds to chorismate mutase and blocks the conversion of chorismate to prephenate, thereby depleting the pool of amino acids required for *M.tb* survival and eventually leading to death of the bacteria within the macrophage.

The probable mechanism of action of DRILS-1398 is summarized in Scheme 1. CM present in the periplasmic space enables the conversion of chorismate to prephenate, which finally results in the production of amino acids phenylalanine and tyrosine, which is utilized for the survival of the *M.tb* within the macrophage. DRILS-1398 binds to CM and blocks the conversion of chorismate to prephenate, thereby depleting the pool of amino acids required for *M.tb* survival and eventually leading to the death of the bacteria within the macrophage.

DRILS-1398 has demonstrated acceptable CYP liabilities, Ames, and hERG inhibitory activities. Toxicity studies in mice were also conducted by repeated dose administration of DRILS-1398 up to 500 mg/kg b.w./day daily once for 7 consecutive days. Histopathological data of twelve vital organs of mice pertaining to both genders revealed no treatment-related adverse effects of DRILS-1398. DRILS-1398 thus emerged as the first inhibitor of *M.tb*-CM that showed efficacy in an *in vivo* mice model. However, further efficacy evaluations of the compound for a month alone and combined with rifampicin are warranted, followed by *in vivo* combination studies in H_37_R_V_ sensitive and MDR strains infected animal models. Further, follow-up regarding combination toxicity studies will be required to ensure the therapeutic potential of DRILS-1398 as an anti-TB drug.

### Limitations of the study

The present study showed that DRILS-1398 is a high-affinity *M.tb*-CM binder. The pharmacological action of DRILS-1398 is possibly mediated through *M.tb*-CM inhibition. However, we do not exclude the possibility of other target engagements, so further investigations are needed. We observed superior efficacy and more of an additive effect when DRILS-1398 is combined with rifampicin *in vitro*. This observation fueled further investigation of DRILS-1398 in combination with different drugs considered in the first-line therapy to enable the preclinical development of the compound. For that, a detailed PK/PD study, efficacy studies and toxicity evaluation are needed in combination with different dosages. While these are our future plans, at this stage, it is beyond the scope of the current study.

## Resource availability

### Lead contact

Information and requests for resources and reagents should be directed to and fulfilled by the lead contact, e-mail: (manojitpal@rediffmail.com).

### Materials availability

All materials used in this study are either commercially available or through collaboration, as indicated.

### Data and code availability

• All data generated or analyzed during this study are included in the present article.
• No new code is generated in this study.
• Any additional information required to reanalyze the data reported in this paper is available from the lead contact upon reasonable request.

## Acknowledgments

Authors thank 10.13039/501100001407Department of Biotechnology, New Delhi, India for financial assistance (grant no. BT/01/COE/07/02, BT/PR12817/COE/34/23/2015 and BT/PR39433/DRUG/134/90/2021). S.C. acknowledges financial support from the 10.13039/501100001843Science and Engineering Research Board (SERB), 10.13039/501100001409Department of Science and Technology, Government of India (grant no. CRG/2021/003804). D.B. thanks DST-INSPIRE, New Delhi, India for a Junior Research Fellowship (IF220618). A.A., a member of Project Implementation Group at Sharda University, thanks Department of Science and Technology, Government of India for DST-FIST facility (TPN-69942) for supporting research. Authors also thank Preclinical Research Department, Anthem Biosciences Pvt. Ltd., Bangalore -560099, India, Department of Safety Assessment, Eurofins Advinus Limited, Bengaluru -560058, India, Foundation for Neglected Disease Research, Bangalore -561203, Karnataka, India for doing some outsourcing work and the Management of DRILS, Hyderabad, India for encouragement and support. S.E.H. is a National Science Chair of the Ministry of Science and Technology, Government of India.

## Author contributions

D.B.: methodology, data curation. R.K.E.: validation, data curation. K.S.K.: methodology and data curation. A.A.: methodology, validation, and data curation. D.K.: validation and data curation. S.C.: software, investigation, data curation, writing – review and editing. G.B.: software, validation, and data curation. F.J.A.: methodology and data curation. F.S.: formal analysis. M.A.: validation and data curation. L.S.: methodology and data curation. G.G.S.: supervision. S.O.: project administration; funding acquisition; and supervision. P.M.: conceptualization; project administration; funding acquisition; and supervision. N.Z.E.: conceptualization; funding acquisition; and supervision. M.P.: conceptualization; project administration; funding acquisition; and supervision. S.E.H.: conceptualization; project administration; funding acquisition; and supervision.

## Declaration of interests

The authors declare no competing interests.

## STAR★Methods

### Key resources table

**Table:** 

REAGENT or RESOURCE	SOURCE	IDENTIFIER
**Bacterial strains**		

*Mycobacterium tuberculosis H37Rv*	ATCC	ATCC 35825
*Mycobacterium tuberculosis H37Rv*	ATCC	ATCC-27294

**Chemicals, peptides, and recombinant proteins**		

Dimethyl sulfoxide	Sigma Aldrich	D8418
Acetonitrile (HPLC grade)	Merck Millipore	34851
CYP 1A2 Inhibitor (Furafylline)	Clearsynth Research Center	CS-T-24960
CYP 2C9 Inhibitor (Sulfaphenazole)	Sigma Aldrich	S0758
CYP 2C19 Inhibitor (Tranylcypromine hydrochloride)	Sigma Aldrich	P8511
CYP 2D6 Inhibitor (Quinidine)	Sigma Aldrich	Q0875
CYP 3A4 Inhibitor (Ketoconazole)	Clearsynth Lab Ltd.	CS-O-11673
EOMCC Substrate (CYP 1A2, 2C19 and 2D6 substrate)	Thermo scientific	P3027
BOMCC Substrate (CYP 2C9 and 3A4 substrate)	Thermo scientific	P2980
Quinidine Sulfate Salt Dihydrate	Sigma Aldrich	Q0875
DPBS	Sigma Aldrich	D8537
Internal buffer solution	Nanion	08 3007
External buffer solution	Nanion	08 3012
EMEM	Sigma Aldrich	M0268
FBS	Gibco	10270
Geneticin (G418)	Thermo Scientific	10131027
Penicillin - streptomycin	Gibco	15140
TrypLE™	Thermo Scientific	12604021
Nano pore chip	Nanion	061104
Tween 80	HiMedia Laboratories Private Limited	GRM225-1
Carboxymethylcellulose sodium salt	Sigma-Aldrich	C5678
K_2_EDTA	HiMedia Laboratories Private Limited	GRM1043
Isoflurane	Raman & Weil Pvt. Ltd.	ISI-20016
Tween 80	Sigma-Aldrich	BCBZ6389
Carboxymethylcellulose sodium salt	Sigma-Aldrich	0000128820
Sodium chloride	RANKEM	J088A21
Sodium phosphate monobasic	RANKEM	J040E21
Sodium Phosphate Dibasic Anhydrous	SRL	7179111
Formaldehyde	Thermo Fisher Scientific	7672460423
Magnesium Sulphate Heptahydrate	RANKEM	J098L22
Ethanol	C & S Chemicals	20211020
Xylene	Thermo Fisher Scientific	7631040323
Acetic acid, glacial	RANKEM	A001A22
DPX Mountant	HIMEDIA	0000561656
Hematoxylin	HIMEDIA	0000591564
Eosin-Y	HIMEDIA	0000548328
Cyanosine	HIMEDIA	0000540234
Sodium bicarbonate	RANKEM	J184C19
2-Propanol	RANKEM	T009A23,T008H23
Dipotassium Ethylenediaminetetraacetic acid	HIMEDIA	0000445401
Heparin, Lithium Salt	Santa Cruz	E0923
Isoflurane USP	Baxter India Private Limited	N023G214A
Fetal Bovine Serum	Gibco	10-082-147
L-glutamine	Sigma-Aldrich	49419
Phorbol 12-myristate 13-acetate (PMA)	Sigma-Aldrich	P1585-1mg
Rifampicin	TCI Chemicals	R0079
RPMI media	Sigma-Aldrich	103360934
Sodium bicarbonate	Sigma-Aldrich	S5761
Sodium Pyruvate	Sigma-Aldrich	P2256
Tissue Culture Grade Water(ultrapure distilled)	HiMedia	TCL 019
Middlebrook 7H10 agar	BD Difco	BD-262710
Middlebrook 7H9 broth	Difco BD	271310
BD Difco™ Bacto™ Dehydrated Agar	Difco BD	214010
Oleic acid-Albumin-Dextrose-Catalase (OADC)	BD	211886
Dimethyl sulfoxide (DMSO)	Sigma -Aldrich	D-4540
Glycerol	Sigma-Aldrich	G-5516
7H11 Agar	BD	90000-526
Isoniazid	Sigma	54-85-3
Tween 20	Sigma	9005-64-5
PEG	Sigma	25322-68-3
L-Tyrosine	SRL	60-18-4
L-Phenylalanine	SRL	63-91-2
L-Threonine	SRL	72-19-5
Pepermint	Sigma Aldrich	8006-90-4
Paraffin Wax	Central drug House (P) Ltd.	184235
ZN acid fast stain Kit	Himedia	K005

**Critical commercial assays**		

Vivid® CYP 1A2 Blue screening kit	Life Technologies	P2863
Vivid® CYP 2C9 Blue screening kit	Life Technologies	P2861
Vivid® CYP 2C19 Blue screening kit	Life Technologies	P2864
Vivid® CYP 2D6 Blue screening kit	Life Technologies	P2972
Vivid® CYP 3A4 Blue screening kit	Life Technologies	P2858
DIMENSION® AST FLEX	Siemens	BA3332
DIMENSION® ALTI FLEX	Siemens	GA4124
DIMENSION® ALPI FLEX	Siemens	GB3315
DIMENSION® TP FLEX	Siemens	FA4068
DIMENSION® ALB FLEX	Siemens	BB4053
DIMENSION® TBI FLEX	Siemens	BB4011
DIMENSION® GLUC FLEX	Siemens	FA3334
DIMENSION® CHOL FLEX	Siemens	FA3259
DIMENSION® CREA FLEX	Siemens	GA4086
DIMENSION® BUN FLEX	Siemens	GC4058
DIMENSION® TGL FLEX	Siemens	GA4041
DIMENSION® PHOS FLEX	Siemens	FD4156
DIMENSION® CA FLEX	Siemens	FA3272
ADVIA EZ Wash	Thermo Fisher Scientific	47406
ADVIA SHEATH RINSE	Thermo Fisher Scientific	72363
ADVIA CBC TIMEPAC	Thermo Fisher Scientific	62244
ADVIA DIFF TIMEPAC	Thermo Fisher Scientific	18362
Electrolyte Ragent Module	Siemens	22051007-192-0201

**Experimental models: Cell lines**		

THP-1 cell lines	ATCC	TIB-202
HEK293 Recombinant Cell line	BPS Bioscience	60619

**Experimental models: Organisms/strains**		

*Mus musculus/ BALB/c*	Vaarunya Biolabs Private Limited	
*Mus musculus/* BALB/c	ICGEB	ICGEB/IAEC/02042019/TACF

**Chemicals, peptides, and recombinant proteins**		

4-Chlorobenzoyl chloride	Sigma-Aldrich	122-01-0
4-Amino-1-methyl-3-N-propyl-1H-pyrazole-5-carboxamide	Chem-Scene	139756-02-8
Triethylamine	Sigma-Aldrich	121-44-8
Dichloromethane	Sigma-Aldrich	75-09-2
Potassium *tert*-butoxide	Sigma-Aldrich	865-47-4
*tert*-Butanol	Sigma-Aldrich	75-65-0
Phosphorus(V) oxychloride	Sigma-Aldrich	10025-87-3
5-Chloroindole	Sigma-Aldrich	17422-32-1
Aluminium chloride	Sigma-Aldrich	7446-70-0
1,2-Dichloroethane	Sigma-Aldrich	107-06-2
4-Chlorobenzoyl chloride	Sigma-Aldrich	122-01-0
Triethylamine	Sigma-Aldrich	121-44-8

**Software and algorithms**		

PyRx	PyRx – Python Prescription	https://pyrx.sourceforge.io/
GROMACS 2020.6	https://www.gromacs.org/^36^^,^^37^	https://manual.gromacs.org/2020.6/download.html

### Experimental model and study participant details

#### Bacterial culture

*Mycobacterium tuberculosis* ATCC- 27294 and *Mycobacterium tuberculosis* ATCC- 35825 were acquired from ATCC. *M.tb* ATCC-35825 is an MDR strain (triple–resistant strain against isoniazid, streptomycin and p-Amino Salicylic acid). Both the *M.tb* strains were grown in Middle brook 7H9 broth (CLSI 2020) and used for experimentation.

#### Mice

Male BALB/c mice aged 8-9 weeks were used for oral pharmacokinetic studies after a minimum of 3 days of acclimatization.^38^^,^^39^^,^^40^ BALB/c male mice, aged 8-12 weeks, weighing 20-25g, were used for the *in vivo* efficacy study. All mice used in this study were maintained at 22°C in a 12/12 hr light-dark cycle in a specific pathogen-free facility and given free access to food and water, and studies were conducted during the light cycle. All the *in vivo* experiments using *M.tb* H_37_Rv infected lab animals were conducted in the BSL3 lab as per the guidelines of the Committee for the Purpose of Control and Supervision of Experiments on Animals (CPCSEA) (www.envfor.nic.in/divisions/awd/cpcsea_laboratory.pdf). BALB/c male mice (8-12 week, 20-25g) were infected with *M.tb* H_37_Rv intratracheally and infection was tested after two days.

#### Study approvals

• The protocols for single-dose oral pharmacokinetic studies were approved by the Institutional Animal Ethics Committee of the Preclinical Research Department, Anthem Biosciences Pvt. Ltd., Bangalore 560099, India (Study no for 50mg/kg PK study: N20084, (ABD/IASE/PR/185/19-22) and the study no for PK studies for 10mg/kg and 30mg/kg of DRILS-1398(F): ABD-8056 and ABD-7967A (ABD/IAEC/PR/89-17-20) respectively.
• The experimental protocol for pharmacokinetics studies in mice protocol was approved by the Institutional Animal Ethics Committee of the Preclinical Research Department, Anthem Biosciences Pvt. Ltd., Bangalore 560099, India (Study no for 10mg/kg IV PK study: ABD-7967B (ABD/IAEC/PR/89-17-20).
• Mutagenesis study (Study no: N20081 dated 01/10/20) was approved by the Preclinical Research Department, Anthem Biosciences Pvt. Ltd., Bangalore 560099, India.
• The protocols for *In vitro* CYP enzyme inhibition study (Study no: N20083 dated 05/10/20) was approved by the Preclinical Research Department, Anthem Biosciences Pvt. Ltd., Bangalore 560099, India.
• The protocol for *In vitro* evaluation of the effect of DRILS-1398 on hERG channel (IKr) over-expressed in human embryonic kidney cells (Study no: N5384 dated 06/11/20 was approved by the Department of Safety Assessment, Eurofins Advinus Limited, Bengaluru -560058, India.
• The *in vivo* efficacy study was approved by Institutional Animal Ethics Committee, International Center for Genetic Engineering and Biotechnology, India (Approval No. ICGEB/IAEC/02042019/TACF-JAMIA-17).
• The protocol for repeated dose 7-day toxicity study of DRILS-1398 was approved by the Institutional Animal Ethics Committee of the Preclinical Research Department, Anthem Biosciences Pvt. Ltd., Bangalore 560099, India. Study no: N23041 (ABD/IAEC/PR/296-23-24).
• The protocol for broth dilution MIC testing against *Mycobacterium tuberculosis* ATCC-35825, by turbidometry in a 96-well microtitre plate format (Study no: S1-EST2324012) was approved by the Foundation for Neglected Disease Research, Bangalore, 561203, Karnataka, India.
• The protocol for determination of the efficacy of DRILS-1398 against intracellular *M.tb* ATCC-27294 and ATCC-35825 in activated THP-1 macrophages (Study no: EST2324039) was approved by the Foundation for Neglected Disease Research, Bangalore, 561203, Karnataka, India.

### Method details

#### Synthesis of DRILS-1398

##### General methods

All of the reactions were carried out under an atmosphere of nitrogen or open-air conditions using oven/flame-dried glassware. All heating reactions were performed in an oil bath. Unless otherwise noted, all commercial reagents and solvents were obtained from the commercial provider and used without further purification. Reactions were monitored by thin layer chromatography (TLC) on silica gel plates (60 F254), visualizing with ultraviolet light or iodine spray. ^1^H and ^13^C NMR spectra were recorded in DMSO-*d*_*6*_ solution by using 400 and 100 MHz spectrometers (VARIAN 400 MR), respectively. Proton chemical shifts (δ) are relative to tetramethylsilane (TMS, δ = 0.00) as internal standard and expressed in ppm. Spin multiplicities are given as s (singlet), d (doublet), t (triplet), m (multiplet), dd = doublet of doublet, as well as bs (broad) etc. Coupling constants (*J*) are given in hertz. Melting points were determined by using melting point apparatus (Buchi melting point B-540) and are uncorrected. MS spectra were obtained on a mass spectrometer (AGILENT 6430 triple quardrupole LC-MS). Chromatographic purity by HPLC (Agilent 1200 series Chem Station software) was determined by using area normalization method and the condition specified in each case: column, mobile phase (range used), flow rate, detection wavelength etc.

##### The steps for preparation of DRILS-1398 as outlined in Figure 1

**Step 1**: The 4-chlorobenzoyl chloride (9.50 g, 54.95 mmol) was added to 4-amino-2-methyl-5-propyl-2*H*-pyrazole-3-carboxylic acid amide (Bell et al. 1992, David et al. 2000) (10 g, 54.94 mM) and triethyl amine (6.94 g, 68.68 mM) in dichloromethane (100 mL), at 0°C. The mixture was stirred for 11-15 h at room temperature. Dichloromethane was removed under a low vacuum, and the mixture was diluted with excess cold water. The white solid was separated, filtered, washed with water (2 × 30 mL), and dried under vacuum to obtain 4-(4-chloro-benzylamino)-2-methyl-5-propyl-2*H*-pyrazole-3-carboxylic acid amide **2** (10.58 g, yield: 60%).

**Step 2:** The mixture of 4-(4-chloro-benzylamino)-2-methyl-5-propyl-2*H*-pyrazole-3-carboxylic acid amide **2** (9 g, 28.11 mmol) and potassium-t-butoxide (9.44 g, 84.33 mmol) in t-butanol (90 mL) was stirred at 90°C, for 20-24 h. After completion of the reaction, the solvent was removed completely under vacuum. The residue was diluted with cold water (50 mL) and acidified with 2N HCl until pH reached 7. The white solid was separated, filtered, washed with excess water, and dried under vacuum to obtain 5-(4-chlorophenyl)-1-methyl-3-propyl-1,6-dihydro-pyrazole(4,3-*d*)pyrimidin-7-one *3* (6.78 g, Yield: 80%).

**Step 3:** A mixture of 5-(4-chlorophenyl)-1-methyl-3-propyl-1,6-dihydro-pyrazole(4,3-*d*)pyrimidin-7-one **3** (4 g, 13.24 mmol) and phosphorous oxychloride (40 mL) was stirred at 100°C, for 12-14 h under anhydrous conditions. After completion of the reaction, the mixture was diluted with cold water with stirring. The white solid separated was filtered, washed with water and dried under vacuum to obtain 7-chloro-5-(4-chlorophenyl)-1-methyl-3-propyl-1*H*-pyrazolo(4,3-*d*)pyrimidine (3.38 g, Yield: 80%).

**Step 4:** A mixture of 7-chloro pyrazolo[4,3-*d*]pyrimidine **4** (1.0 equiv), 5-chloroindole (1.0 equiv) and AlCl_3_ (1.2 equiv) in dichloroethane (5 mL) were stirred at 80°C for 7 h under nitrogen. After completion of the reaction, the mixture was poured into ice-cold water (15 mL), stirred for 10 min and then extracted with excess of ethyl acetate. The organic layers were collected, combined, washed with cold water, dried over anhydrous Na_2_SO_4_, filtered and concentrated under vacuum. The residue obtained was purified by column chromatography on silica gel using ethyl acetate/hexane to obtain the desired product.

#### Molecular modeling studies

##### *In-silico* docking studies

The 3-D structure of DRILS-1398 was prepared and optimized using the quantum mechanical method with the 6-31G(d) basis set. The optimized ligand was then prepared for docking by merging non-polar atoms, assigning charges to the atoms, and defining the rotatable bonds.

Crystal structures of secretory dimeric chorismate mutase (CM) from *Mycobacterium tuberculosis* H_37_Rv were available from the protein data bank (PDB IDs: 2AO2,^41^ 2F6L,^32^ and 2FP2.^42^ Structural alignments revealed minimum structural deviations among them. Thus, the crystal structure of apo CM (2F6L, resolution = 1.7 A) was considered for further study. Notably, the recently available AlphaFold structure of CM also showed perfect alignment of the chorismate mutase domain to the apo crystal structure (RMSD < 0.3 A). Thus, the apo crystal structure of the CM (PDB ID: 2F6L) was considered further for molecular modeling studies.

The ligand binding sites of dimeric CM were then identified using the PrankWeb^33^ webserver. We identified three binding pockets: two are in each CM domain, and the other is at the interface between the two domains. All three binding sites were considered separately during the molecular docking. Each binding site was then probed by selecting the appropriate grid box covering the particular site only and prepared for docking using the PyRx program. The molecular docking of the ligand to each ligand binding site of CM was performed using AutoDock Vina^43^ using the PyRx virtual screening tool (https://sourceforge.net/projects/pyrx/). The lowest energy docked conformations of the ligand on two different binding sites (among three sites) exhibited comparable docking scores. Thus, these two differently docked complexes were further studied using extensive molecular dynamics simulations.

##### Molecular dynamics simulations

All the simulations were performed using the GROMACS 2020.6 packages.^36^^,^^37^ Amber99sb-ILDN forcefield^44^ with TIP3P water model^45^ used to simulate apo CM and CM-ligand complexes. The optimized ligand structure was used to parameterize the ligand according to the general Amber force field (GAFF) using Antechamber tools.^46^^,^^47^ The parameter sets were made compatible with the GROMACS package using the ACPYPE program.^48^ The apo-protein and two differently docked complexes were then placed in a cubic box, and box dimensions were chosen so that the distance between any protein atom and box edges was > 10 Å. All the systems were solvated with TIP3P water and made charge neutral by adding 11 Na^+^ ions to each system. All the systems were made periodic in all three dimensions, and then energy was minimized using the steepest descent algorithm for 500 steps. All the minimized systems were subjected to a series of equilibration simulations, including 200 ps position-restrained dynamics in the NVT ensemble, followed by 2 ns of unrestrained dynamics in the NVT ensemble and then subsequently in the NPT ensemble. Finally, production simulations were carried out on each equilibrated system for 1 μs in the NPT ensemble at 298 K and 1 bar. The temperature was maintained at 298 K by coupling with a Nose–Hoover thermostat using a coupling time constant of 0.1 ps, and pressure was maintained by coupling with a Parrinello–Rahman isotropic barostat with a coupling time constant set to 2 ps. The particle mesh Ewald summation method^49^ was used to calculate the electrostatic interactions.

#### Cloning, expression and purification of chorismate mutase enzyme

Rv1885C, encoding *M.tb* Chorismate Mutase (CM), was PCR amplified from *M.tb* genomic DNA using the primers (Fwd: 5’TGGATCCTTGCTTACCCGTCCACGTGAGATATA3’ and Rev: 5’AATAAGCTTGGCCGGTAGGGCCTGGCAA3’). The amplification conditions were as follows: Initial denaturation of DNA at 94° for 5 min. This was followed by 2 amplification cycles- Denaturation at 94°C for 30 sec, annealing at 50°C for 30 sec and extension at 72°C for 60 sec for a total of 10 cycles. Next cycle was carried out at -denaturation at 94°C for 30 sec, annealing at 60°C for 30 sec and extension at 72°C for 60 sec for 30 cycles. Final extension was carried out at 72°C for 10 min The amplified DNA was cloned into pET15b between the sites Nde1 and Xho1 and the recombinant DNA was isolated from E. coli DH5α.

The PET15b recombinant was transformed in BL21∗DE3 for the production of *M.tb*-CM. E. coli BL21∗DE3 harboring the recombinant plasmid was grown in LB medium supplemented with ampicillin (100μg/mL) at 37°C to an A_260_ of _∼_0.6. *M.tb* CM was induced with 30 μM IPTG and kept for incubation at 25°C overnight.

For the purification of *M.tb*-CM, the culture was scaled up to 1 liter, and the cells were harvested by centrifugation at 4000 rpm at 4° for 15 minutes. The pellet was resuspended in 10 mL of 20% sucrose and 30mM Tris-HCl (pH 8.0) and 1 M EDTA was added to a final concentration of 1 mM. The suspension was mixed on a rotary shaker at 150 rpm for 10 min at 25°C and centrifuged at 13000rpm for 15 min at 4°C. The cell pellet was suspended in 4 mL of ice-cold 5 mM MgSO_4_ and kept on a rotary shaker at 150 rpm for 10 min at 25°C. The cell suspension was centrifuged at 13000 rpm for 15 min at 4°C. The supernatant containing the periplasmic proteins was collected and buffered with 1M Tris pH8.0 to a final concentration of 30 mM. The Periplasmic fluid was concentrated to ∼1ml and injected into Mono Q 5/50 GL prepacked anionic exchanger FPLC Column. The column was equilibrated with 30mM Tris pH 8.0 and 5mM DTT and the protein was eluted with 30mM TRIS pH 8.0, 5mM DTT and 250 mM NaCl. The purified CM was quantified using Bradford, and the 100 μL aliquots were preserved at -80°C^32^ (Figure S15).

#### CM assay

The activity of the CM enzyme was based on direct observation of the conversion of chorismate to prephenate at OD 274 nm.^32^ The reaction volume of the assay was maintained at 100 μl. 1mM of the substrate, chorismic acid (Sigma) was pre-incubated at 37°C for 5 min in the buffer (containing 50mM Tris-HCl (pH 7.5), 0.5mM EDTA, 0.1 mg/mL bovine serum albumin, and 10 mM β-Mercaptoethanol). The reaction was started by adding 180 pmol of CM enzyme to the prewarmed chorismic acid solution. Inhibitory screening of the compounds against CM activity was measured at 100 nM to 50 μM concentration of the effectors. The reaction was allowed to proceed at 37°C and was terminated after 5 min with 100 μl of 1 N HCl. Disappearance of substrate was measured at 274 nm. A blank with no enzyme for every reaction was kept as a control to account for the non-enzymatic conversion of chorismate to prephenate.

#### Protease protection assay protocol

3 μg of purified recombinant *M.tb* Chorismate mutase was incubated with the compound at 10 μM and 30 μM for 20 min. Trypsin was added to a final concentration of 400 μg/mL and incubated for 5 mins. The reaction was terminated with 5x Gel loading dye and resolved in 18% SDS-PAGE.

#### Broth dilution MIC testing against *Mycobacterium tuberculosis* ATCC-35825, by turbidometry in a 96-well microtitre plate format

*M.tb* ATCC- 35825 is an MDR strain (triple–resistant strain against isoniazid, streptomycin and p-amino salicylic acid). *Mycobacterium tuberculosis* ATCC- 35825 was grown in Middle brook 7H9 broth (CLSI 2020). The test compounds diluted in DMSO were tested in triplicate. Test compounds were assayed at a 256 μg/mL start concentration and 2-fold serially diluted in a 10-concentration dose response. A volume of 4 μL of respective dilutions of compounds was then transferred to the assay plate. To the assay plate wells containing test compounds, *Mycobacterium tuberculosis* H_37_Rv (ATCC-35825) culture inoculum of 200 μl was added (strength of 3-7X10^5^ cfu/mL). The assay plates were then incubated at 37°C/14.^35^ This work (Study no: S1-EST2324012) was approved by the Foundation for Neglected Disease Research, Bangalore, 561203, Karnataka, India.

#### Determination of the efficacy of DRILS-1398 against intracellular *M.tb* ATCC-27294 and ATCC-35825 in activated THP-1 macrophages

The THP-1 cells were activated with PMA (phorbol 12-myristate-13-acetate) for differentiation into macrophages. After 72 hr of activation, the THP-1 macrophages were infected with *M.tb* H_37_Rv strain (ATCC-27294 and ATCC-35825) at a multiplicity of infection (MOI) of 1:10.^34^^,^^35^ The macrophage infection was allowed for 2 hrs at 37°C with 5% CO_2_. The media containing *M.tb* was discarded, and macrophage monolayers were washed twice with phosphate-buffered saline to remove the extracellular bacteria and replenished with fresh, complete RPMI. The *M.tb*-infected macrophages were exposed to compounds by transferring 200 *μ*L of fresh media containing the respective concentrations of the compounds. Duplicate wells were lysed (0.05% SDS) at specific time points and enumerated to estimate the numbers of intracellular *M.tb* 2 hr post-infection. The 0.1 mL of lysate was serially diluted and plated onto Middle brook 7H11 agar plates for enumeration (read after 3 to 4 weeks) of live *M.tb* after compound treatment. After 3 and 7 day exposure to the compounds, the cells were treated the same way, and the live *M.tb* was enumerated. The efficacy of the test compound was assessed by comparing the *M.tb* counts from the respective days to day 0 and day 7. The same protocols were followed with doses for the combination studies in both strains, as indicated in the legend of Figure 3. This work (Study no: EST2324039) was approved by the Foundation for Neglected Disease Research, Bangalore, 561203, Karnataka, India.

#### Experimental protocol for mice microsomal stability study

The objective of the study was to perform a metabolic stability study for DRILS-1398 at 3.0 μM test concentration in mice liver microsomes (0.25 mg/mL) and to calculate the half-life (t_1/2_, min) and intrinsic clearance (CL, μL/min/mg). The metabolic stability study prepared the metabolic reaction mixture for DRILS-1398 at 3.0 μM (0.1% DMSO) in mice liver microsomes in the presence and absence of cofactors (NADPH regeneration system). The prepared metabolic reaction was incubated at 37°C, 400 rpm for 2 hr using Thermomixer. At specified incubation time points (with cofactor-0.0, 15, 30, 60, and 120 min; without cofactor-0.0 and 120 min), 100 μL of the reaction mixture was collected and subjected to sample extraction. LC-MS/MS analysis was performed on the extracted samples to determine the amount of DRILS-1398 remaining compared to 0.0 min in the presence of cofactors.^50^^,^^51^

#### Experimental protocol for protein binding study

DRILS-1398 (5μM) was added to both sides of the membrane in an equilibrium dialysis system. Species-specific plasma (50% or 100%) was added to one side, and the system was allowed to reach equilibrium at 37^o^C. LC-MS/MS measurements of compound concentration were performed on both sides of the membrane and the fraction of unbound compound was presented.^52^

#### Development of formulation of DRILS-1398

The following approaches were attempted to develop an appropriate formulation of DRILS-1398.

(i) Development of nanoemulsion using (a) DRILS-1398, peppermint oil, Tween 80: PEG 400 (as surfactant: co-surfactant) and normal saline (0.9% w/v) (as an aqueous phase); and (b) DRILS-1398, peppermint oil, Tween 20: PEG 200 (as surfactant: co-surfactant) and normal saline (0.9% w/v) (as an aqueous phase).
(ii) Development of micelles using DRILS-1398, PEG 400: Tween 80 (as surfactant: co-surfactant) and normal saline (0.9% w/v) (as an aqueous phase).

The second approach was found to be promising and, therefore, was explored further. After assessing various micelles, it was observed that the following composition, i.e. 0.2-0.9 % DRILS-1398, 10-30% Smix containing tween 80: PEG 400 (1:1) and 70-90% normal saline, was found to be most appropriate. More particularly, the following formulation, i.e., 0.3% DRILS-1398, 20% S mix containing tween 80: PEG 400 (1:1), and 79.7% normal saline, was preferred for pharmacological studies.

#### Single-dose oral pharmacokinetic studies of DRILS-1398 in male BALB/c mice at a dose of 50 mg/kg body weight and formulated DRILS-1398(F) at doses of 10 mg/kg and 30 mg/kg body weight

Male BALB/c mice aged 8-9 weeks were used for experimentation after a minimum of 3 days of acclimatization.^38^^,^^39^^,^^40^ Fasted animals were administered with DRILS-1398 or DRILS-1398 (F) in a recommended vehicle (1%(v/v) Tween 80 + 99% (v/v) of 0.5% (w/v) Carboxymethylcellulose sodium salt in ultrapure water) by oral route with a dose of 50 mg/kg body weight of DRILS-1398 or 10 mg/kg and 30 mg/kg for DRILS-1398(F) at a dose volume of 10 mL/kg b.w. Under mild isoflurane anesthesia, a blood specimen was collected into pre-labeled tubes containing anticoagulant (K_2_EDTA - 2 mg/mL blood) during the next 24 hours of post-dose (Table-PK study design chart). The collected blood specimens were centrifuged at 6000 rpm, 4°C for 10 min, and plasma was separated and stored at -80°C until analysis. All animals were observed for any apparent signs of toxicity related to the dose or test item during the study period. Concentrations of analyte DRILS-1398 in mice plasma samples were determined using the Shimadzu LCMS-8045 LC-MS/MS system. The protocols were approved by the Institutional Animal Ethics Committee of the Preclinical Research Department, Anthem Biosciences Pvt. Ltd., Bangalore 560099, India (Study no for 50mg/kg PK study: N20084, (ABD/IASE/PR/185/19-22) and the study no for PK studies for 10mg/kg and 30mg/kg of DRILS-1398(F): ABD-8056 and ABD-7967A (ABD/IAEC/PR/89-17-20) respectively.

**Table:** **Table-PK study design chart** : Experimental design for single-dose oral PK Study of DRILS-1398 and DRILS-1398 (F) in Male BALB/c Mice. G indicates different study group.

Group No.	Animal ID	Blood (X) Collection time points (h)									
		0.08	0.16	0.25	0.50	1.00	2.00	4.00	6.00	8.00	24.00
G-1	M0001	X	-	-	-	-	X	-	-	-	-
	M0002	X	-	-	-	-	X	-	-	-	-
	M0003	X	-	-	-	-	X	-	-	-	-
G-2	M0004	-	X	-	-	-	-	X	-	-	-
	M0005	-	X	-	-	-	-	X	-	-	-
	M0006	-	X	-	-	-	-	X	-	-	-
G-3	M0007	-	-	X	-	-	-	-	X	-	-
	M0008	-	-	X	-	-	-	-	X	-	-
	M0009	-	-	X	-	-	-	-	X	-	-
G-4	M0010	-	-	-	X	-	-	-	-	X	-
	M0011	-	-	-	X	-	-	-	-	X	-
	M0012	-	-	-	X	-	-	-	-	X	-
G-5	M0013	-	-	-	-	X	-	-	-	-	X
	M0014	-	-	-	-	X	-	-	-	-	X
	M0015	-	-	-	-	X	-	-	-	-	X

#### Experimental protocol for pharmacokinetics studies in mice (IV: Intravenous)

The intravenous (IV) (10 mg/kg of DRILS-1398) dosing study on BALB/c male mice (n=3 per time point, not more than 2 bleeds per animal, total animals dosed n=12) was carried out on the following time points through saphenous veins; IV: 15 mins, 30 mins, 1, 2, 4, 6, 8 and 24 hr. Collected blood specimens were centrifuged at 6000rpm, 4°C for 10 minutes, and plasma was separated and stored at -80°C until analysis. All animals were observed for any apparent signs of toxicity related to the dose or test item during the study period. Concentrations of analyte DRILS-1398 in mice plasma samples were determined using the Shimadzu LCMS-8045 LC-MS/MS system. The protocol was approved by the Institutional Animal Ethics Committee of the Preclinical Research Department, Anthem Biosciences Pvt. Ltd., Bangalore 560099, India (protocol number for 10 mg/kg IV PK study: ABD-7967B (ABD/IAEC/PR/89-17-20).

#### Mutagenesis study

The study aimed to evaluate the potential mutagenic activity of the test item DRILS-1398 as per the OECD 471, Guideline for the Testing of Chemicals: Bacterial Reverse Mutation Test, by examining its ability to revert three histidine-dependent strains of Salmonella typhimurium - TA98, TA100 and TA1537 - in the absence and presence of a rat liver S9 metabolizing system (referred here onwards as S9) with Standard Plate Incorporation (SPI) method.^53^^,^^54^^,^^55^ As a part of the solubility check, DRILS-1398 was soluble in DMSO at 50 mg/mL. However, precipitation was observed with top agar at 50 and 40 mg/mL. 3000 μg/plate (100 μL aliquot per plate from 30 mg/mL stock solution) was chosen as the highest concentration for the preliminary cytotoxicity study. A Mutagenicity factor compared the number of revertant colonies in the vehicle was calculated using Microsoft Excel. This work (Study no: N20081 dated 01/10/20) was approved by the Preclinical Research Department, Anthem Biosciences Pvt. Ltd., Bangalore 560099, India.

#### *In vitro* CYP enzyme inhibition study of DRILS-1398 by fluorescent substrate method

This study performed CYP inhibition assay using Vivid CYP P450 (1A2, 2C9, 2C19, 2D6, and 3A4) blue screening kits.^56^ The test item DRILS-1398 was incubated at eight different test concentrations (30, 10, 3, 1, 0.3, 0.1, 0.03, and 0.01 μM in 1% acetonitrile). Furafylline (CYP 1A2 inhibitor), Sulfaphenazole (CYP 2C9 inhibitor), Tranylcypromine (CYP 2C19 inhibitor), Quinidine (CYP 2D6 inhibitor), Ketoconazole (CYP 3A4 inhibitor) were used as positive controls for specific CYP enzymes. The fluorescent reading was taken using a microplate reader at excitation/emission wavelengths of 415/460 nm, respectively. The % of CYP inhibition of test/positive control at each tested concentration was calculated, and the IC_50_ value against each CYP isoform was plotted using Graph pad prism software. This work (Study no: N20083 dated 05/10/20) was approved by the Preclinical Research Department, Anthem Biosciences Pvt. Ltd., Bangalore 560099, India.

#### *In vitro* evaluation of the effect of DRILS-1398 on hERG channel (IKr) over-expressed in human embryonic kidney cells

Patch clamp experiments were carried out using a Port-a-Patch system (Make: Nanion), which automatically obtains Giga ohm seals (Guidance for industry: S7B: The Nonclinical Evaluation of the Potential for Delayed Ventricular Repolarization (QT Interval Prolongation) By Human Pharmaceuticals. ICH May 2005). The entire process was software-controlled and semi-automated. This protocol (Study no: N5384 dated 06/11/20 was approved by the Department of Safety Assessment, Eurofins Advinus Limited, Bengaluru -560058, India.

HEK cells stably transfected with the hERG gene were maintained at 37±2°C in a 5% CO_2_ incubator. The selection medium in the flask was removed one day before the experiment, and cells were refreshed with a complete growth medium and maintained at 37±2°C in a 5% CO_2_ incubator. On the day of the experiment, cells were observed under a microscope for confluency, and cells with a confluency of 70 - 80% were used. The culture medium from the flask was removed, and cells were washed with 2 mL of DPBS, aspirated and discarded. The cells were then treated with 1 mL of the TrypLETM (cell dissociation solution) and incubated for 2 mins at 37°C in a CO_2_ incubator. A volume of 5 mL of complete growth medium was added to the flask, and the cells were dislodged from the flask by gently pipetting up and down. The cell suspension was transferred into a sterile test tube, and the cells were centrifuged at 1000 rpm for 5 min at room temperature. The supernatant was removed, and the cells were resuspended in an external buffer solution. All the experiments were performed at ambient temperature.

##### General experimental procedure using Port A patch

The NPC-1 chip of the Port-a-Patch was filled with 5μL of internal buffer solution and it was screwed onto the chip holder. The Faraday cage was then fixed on the chip holder such that the external electrode was placed near the chip aperture. 5 μL of external buffer solution was added to the center of the aperture so that the external electrode also comes in contact with the external buffer solution. The experiment was then initiated by clicking the play button. Once the threshold resistance of NPC chip was attained (i.e., 2 - 3.5 MOhm), 5 μL of cell suspension was added into the middle of the external buffer solution droplet after the suction control unit had generated the suction pulse. The suction automatically pulls a cell to the aperture, resulting in an increase in the chip resistance. When the cell was caught and the set threshold for the resistance reached (i.e. 5 MOhm), the software recognizes this increase and proceeds to the next step of the sealing procedure. 20 μL of seal enhancer solution was then added after 2-3 washes. Patch Control automatically recognizes the resistance reaching the threshold and moves to the next steps of improving the seal. When the desirable resistance (Rpip) was reached, whole-cell configuration was attained and maintained. The hERG pulse protocol was selected to measure the current amplitude resulting from the exchange of ions through the hERG channel. The seal enhancer solution was exchanged with 20 μL external solution after 2-3 washes.

##### A schematic diagram of the pulse protocol

Cells were stimulated every 10 seconds using the following protocol. The holding potential, sweep intervals, and depolarization/repolarization potentials are furnished in (Figure S16).

##### Test/control item exposure

Once the stable current was achieved, the steady state current phase with vehicle control was considered as a control reading. The vehicle control was replaced with 20 μL of test item concentrations from lower to higher concentrations (0.1 and 30 μM) each after 2-3 washes. After adding each test item, the concentration current was measured for 3-5 minutes. The steady-state current phase with each test concentration was considered as the current reading for that particular concentration. Once all the concentrations were tested, Propafenone hydrochloride (10 μM) was added to the cell after 2-3 washes and the current was measured for 3 -5 minutes to check for the sensitivity of the test system. All the test concentrations and positive control were tested in triplicates.

##### Data compilation

The experimental data points were exported in the form of ∗.asc files with experiment number, series number, concentrations tested and date of experiment in a separate folder with the respective study number. This file was then imported to MS excel sheets for analysis. The average of 3-5 data points (stable data points without fluctuations) among the last 10 data points of vehicle control current was considered as control reading for comparison/calculation. Similarly, the average of stable 3-5 data points among last 10 data points of test item concentrations / positive control reading was considered for calculation and reporting. The percentage inhibition by test concentration and positive control was calculated relative to the control reading.

#### *In vivo* efficacy study

All the *in vivo* experiments using *M.tb* H_37_Rv infected lab animals were conducted in BSL3 lab as per the guidelines of the Committee for the Purpose of Control and Supervision of Experiments on Animals (CPCSEA) (www.envfor.nic.in/divisions/awd/cpcsea_laboratory.pdf). The experiment protocols were approved by Institutional Animal Ethics Committee, International Center for Genetic Engineering and Biotechnology, India (Approval No. ICGEB/IAEC/02042019/TACF-JAMIA-17). BALB/c male mice (Male, 8-12 weeks, 20-25g) were infected with *M.tb* H_37_Rv, and clearance of infection was assessed in the lungs and spleen.^57^ Mice were infected intratracheally with *M.tb* H_37_Rv and infection was tested after two days. After four weeks of infection, mice (n=6) were sacrificed, and lungs and spleen were excised, homogenized and plated on 7H11-OADC agar plates. Mice showed distinct granuloma in the lungs, which was distinctive of *M.tb* infection load. After the fourth week of infection, different groups of control and *M.tb* H_37_Rv infected mice were administered drugs on alternate days. The following control and treatment groups of mice were examined:

Group 1: Uninfected

Group 2: Infected with *M.tb* H_37_Rv

Group 3: Treated with DRILS- 1398 (10-50 mg/Kg)

Group 4: Treated with DRILS-1398(F) (10-50 mg/Kg)

Group 5: Treated with Isoniazid (5mg/Kg)

Group 6: Treated with surfactant (Tween + PEG)

Drug treatment: Once in two days/alternative day

##### Histopathology analysis

The lung samples obtained from the control and infected group of mice were immersed in 10 % formalin. Paraffin block of the tissue sample was prepared, 6-μm-thick histological sections were obtained with a rotary microtome (Spencer microtome). Samples were stained with hematoxylin and eosin, then examined under a light microscope (Carl Zeiss). Detailed analysis of the specimen for histological analysis of control and drug treatment tissue of infected mice was carried out and representative micrographs (4x and 10x magnifications) were obtained. Ziehl-Neelsen (ZN) staining was done to assess the presence of acid-fast bacilli within the lung tissue sample and micrographs (40x magnification) were obtained. Pathologist carried out blind evaluation of the samples.

#### Repeated dose 7-day toxicity study of DRILS-1398

As per the guideline,^58^^,^^59^ 40 males and 40 females were assigned into four groups of ten animals/sex per group viz., Vehicle control (G1), Low dose (G2), Mid Dose (G3) and High dose (G4). The test item formulations were prepared freshly in Ultra-pure water type 1 as a vehicle. The vehicle alone was administered to the vehicle control (G1) animals. Test item formulations were administered daily once at dose levels of 125 (G2), 250 (G3) and 500 (G4) mg/kg b.w./day. All the animals were administered a dose volume of 10 mL/kg b.w. once daily for 7 consecutive days. All the animals were observed once daily for clinical signs, twice daily for mortality/morbidity, and on day 7 for detailed clinical examination. Body weights and feed consumption were recorded at weekly intervals. At the end of the experimental period (day 8), blood and harvested plasma specimens were analyzed for haematology and clinical chemistry parameters, respectively. Subsequently, the animals were euthanized and subjected to gross pathological examination. The specified organs were collected and preserved in 10% NBF for histopathological evaluation. The protocol was approved by the Institutional Animal Ethics Committee of the Preclinical Research Department, Anthem Biosciences Pvt. Ltd., Bangalore 560099, India. Study no: N23041 (ABD/IAEC/PR/296-23-24).

### Quantification and statistical analysis

The statistical significance of CFU in Figure 5 was determined by two-way ANOVA. Values were expressed as mean ± S.D. p< 0.05 was considered significant, ∗p < 0.05, ∗∗p < 0.01, ∗∗∗p < 0.001.

Detailed analysis of log_10_ cfu reduction and its statistical significance for each combination dosage against *M.tb-*H_37_R_v_ strain ATCC 27294 and a multi-drug resistant strain ATCC 35825 are shown in supplementary figures, Figures S8 and S9, respectively. Values were expressed as mean ±SEM. Statistical significance was calculated using GraphPad Prism. For comparison between 2 groups, the unpaired Student’s t-test was used. One-way ANOVA followed by Bonferroni’s post hoc analysis or Dunnett’s post hoc analysis. p< 0.05 was considered significant, ∗p < 0.05, ∗∗p < 0.01, ∗∗∗p < 0.001.
