# Supplementary material for: Early preclinical development of Mycobacteriumtuberculosis amino acid biosynthesis pathway inhibitor DRILS-1398 as a potential anti-TB drug
Source: iScience. 2025 Apr 29;28(6):112537. doi: 10.1016/j.isci.2025.112537 (PMC12144419; doi:10.1016/j.isci.2025.112537)
Supplement: Document S1. Figures S1–S16 and Tables S1–S4 [file mmc1.pdf]

iScience, Volume *28*

Supplemental information

Early preclinical development of *Mycobacterium tuberculosis* amino acid biosynthesis pathway inhibitor DRILS-1398 as a potential anti-TB drug

Deepesh Biswas, Rebecca Kristina Edwin, K. Shiva Kumar, Anwar Alam, Dhiraj Kumar, Sandipan Chakraborty, Gopalakrishnan Bulusu, Farhan Jalees Ahmad, Gautham G. Shenoy, Lakshyaveer Singh, Mansi Agarwal, Fouzia Siraj, Srinivas Oruganti, Parimal Misra, Nasreen Zafar Ehtesham, Manojit Pal, and Seyed Ehtesham Hasnain

Supplemental information:

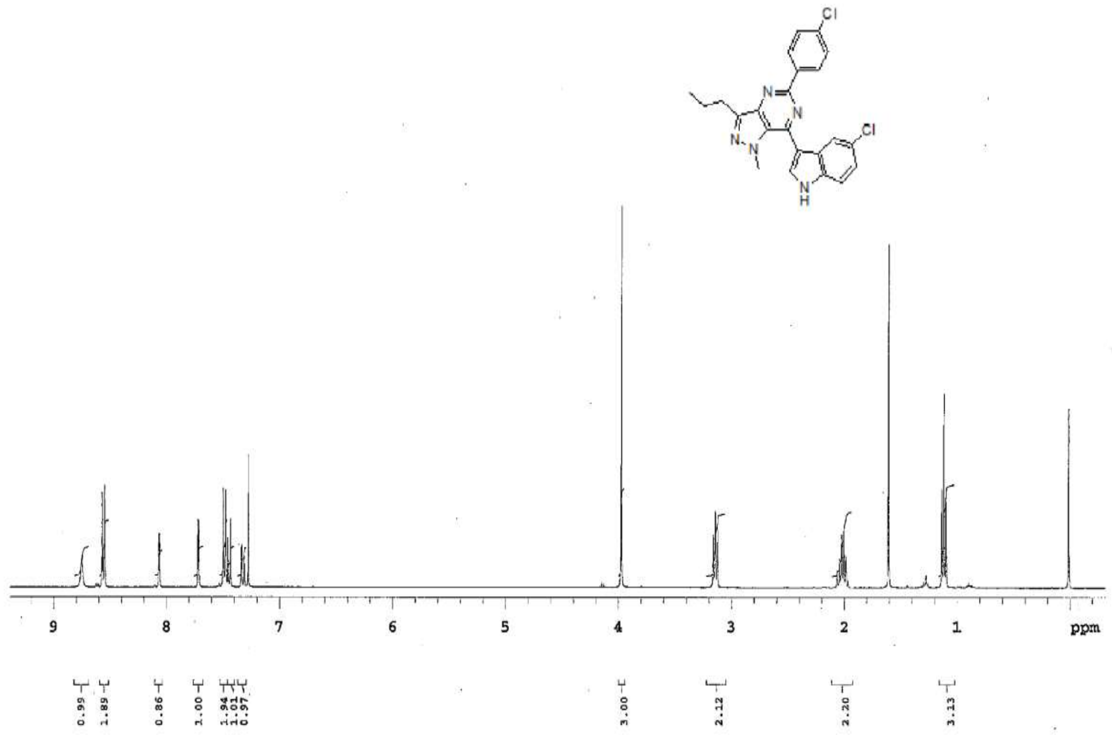

Fig. S1: ^{1}H NMR (400 MHz, $CDCl_3$) spectra of DRILS-1398, related to Fig. 1.

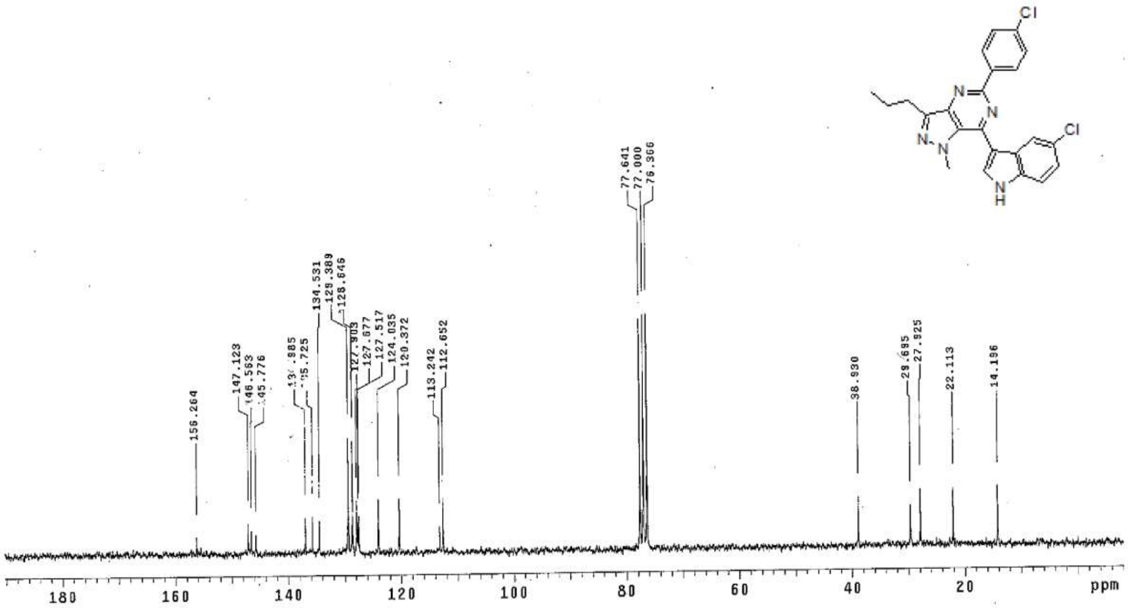

Fig. S2: ^{13}C NMR (100 MHz, $CDCl_3$) spectra of DRILS-1398, related to Fig. 1.

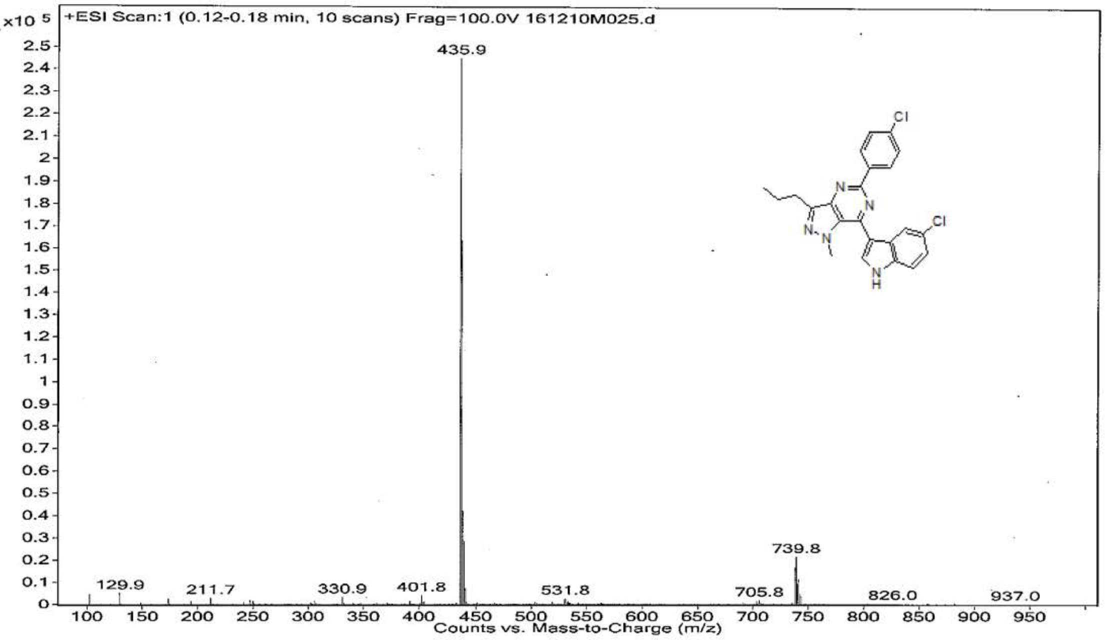

Fig S3: Mass spectra of DRILS-1398, related to Fig. 1.

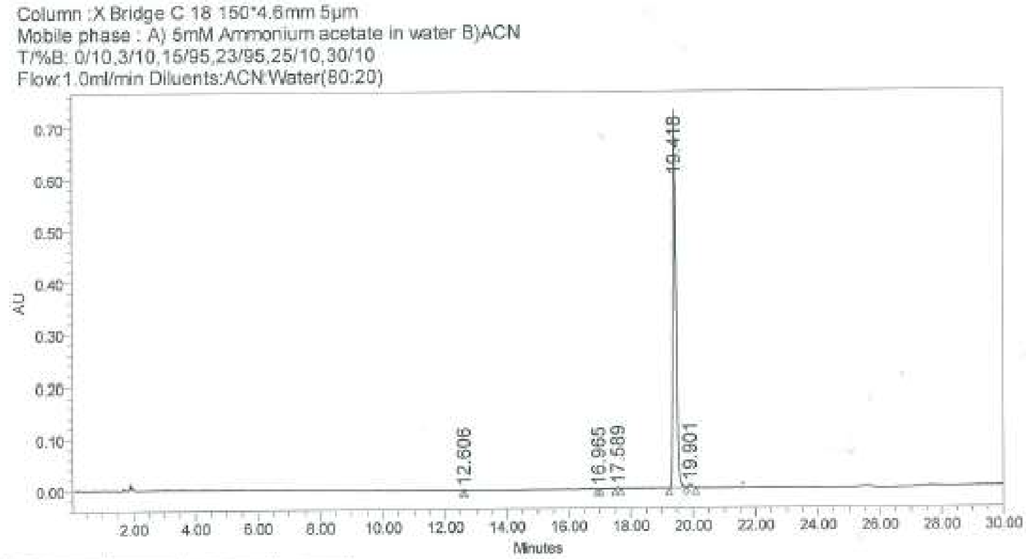

	RT	Height	Area	% Area
1	12.606	1622	4805	0.09
2	16.965	329	1174	0.02
3	17.589	2289	9075	0.18
4	19.418	728757	5044952	98.57
5	19.901	7687	58143	1.14

Fig S4: Detection of DRILS-1398 using HPLC, related to Fig. 1.

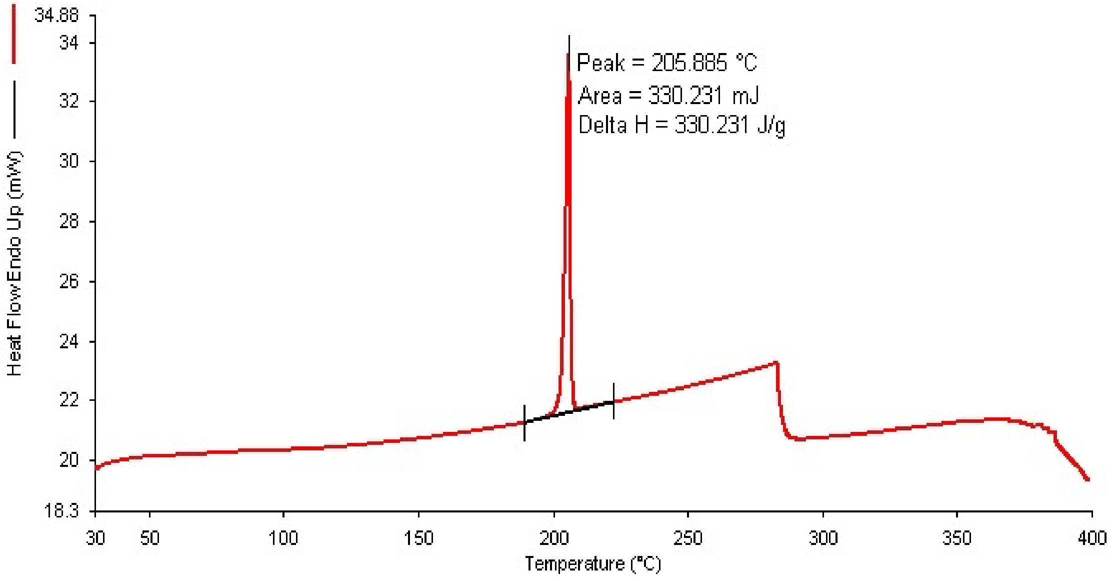

Fig. S5: DSC Curve of DRILS-1398, related to Fig. 1.

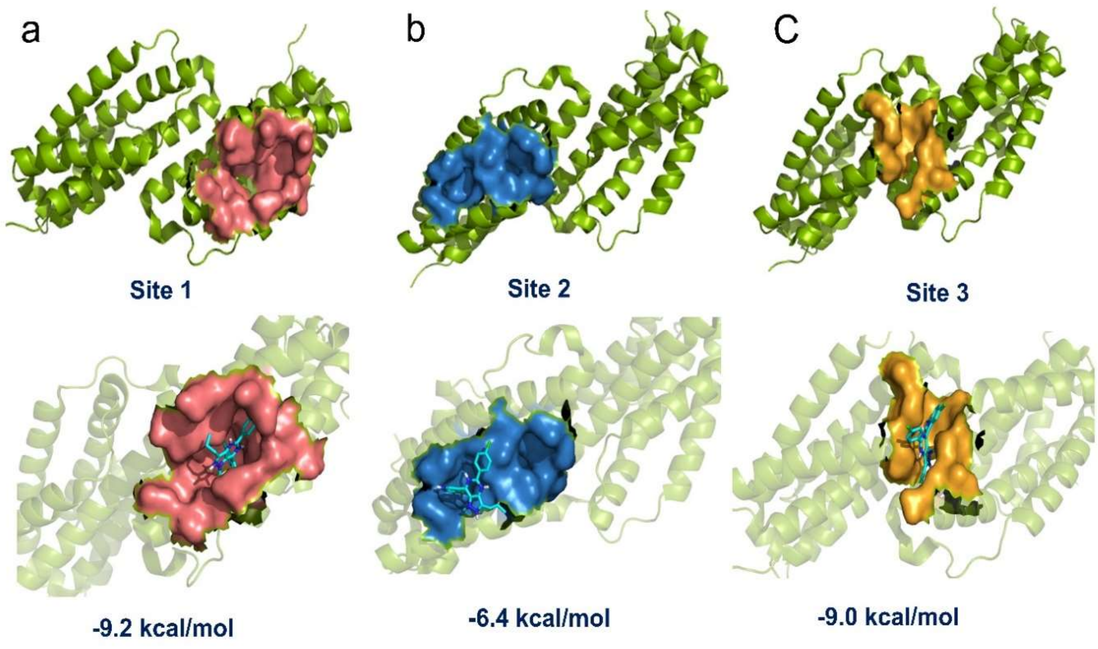

Fig. S6: Representation of possible binding sites of chorismate mutase, related to Fig. 2. Site 1 (A), Site 2 (B), and Site 3 (C) are shown in pink, blue, and orange surface representation, respectively. The lowest energy docked complexes of DRILS-1398 (cyan stick) at each binding site are shown in the lower panel.

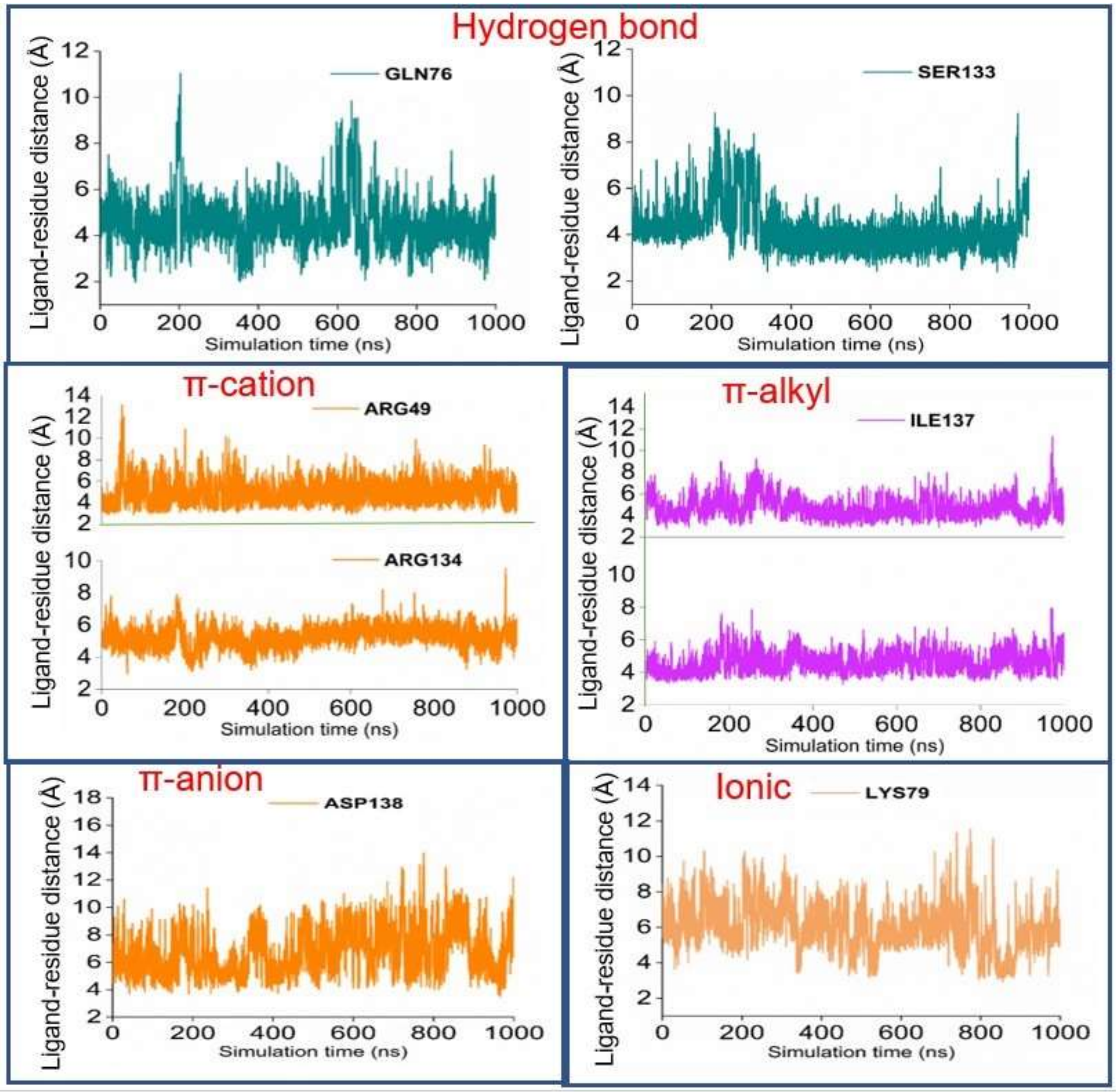

Fig. S7: The stability of the dominant protein-ligand interactions during the 1 µs MD simulation trajectory, related to Fig. 2.

Efficacy of DRILS-1398 in combination with Rifampicin against intracellular *M. tuberculosis* H37Rv ATCC 27294 in activated THP-1 macrophages

Cell lines	Activated THP-1 macrophages	
Mtb strain	*M. tuberculosis* H37Rv ATCC 27294	
MOI	THP-1: Mtb = 1:10	
Combinations tested	Test compound	DRILS-1398: 15, 7.5, 3.75, 1.875, 0.93µM
	Assay controls	Rifampicin: 19.45µM
Results		

M. tuberculosis H37Rv	Drugs used in combination (µM)		*Log₁₀ cfu drop vs. D0 Control*		Log_{10} cfu drop vs. D3 Control	Significance	Log_{10} cfu drop vs. D7 Control	Significance
	RIF	DRILS-1398	Day 3	Day 7	Day 3		Day 7	
Combination 1	19.45	15	1.27	2.37	1.55	***	2.99	****
Combination 2	19.45	7.5	0.96	1.99	1.24	***	2.61	***
Combination 3	19.45	3.75	0.94	1.89	1.22	***	2.5	***
Combination 4	19.45	1.8	0.94	1.72	1.21	***	2.33	***
Combination 5	19.45	0.93	0.93	1.61	1.21	***	2.23	***
Drugs alone	19.45	-	0.98	1.66	1.26	***	2.28	***
	-	15	0.15	0.36	0.42	**	0.98	**
Cell control			-0.28	-0.62				

Fig. S8: Efficacy of DRILS-1398 in combination with Rifampicin against intracellular *M. tuberculosis* $H_{37}R_{v}$ ATCC 27294 in activated THP-1 macrophages, related to Fig. 4. A detailed analysis of Log_{10} cfu reduction and its statistical significance for each combination dosage are shown.

Efficacy of DRILS-1398 in combination with Rifampicin against intracellular *M. tuberculosis* ATCC 35825 in activated THP-1 macrophages

Cell lines	Activated THP-1 macrophages	
Mtb strain	*M. tuberculosis* ATCC 35825	
MOI	THP-1: Mtb = 1:10	
Combinations tested	Test compound	DRILS-1398: 15, 7.5, 3.75, 1.8, 0.93µM
	Assay control	Rifampicin: 19.45µM
Results		

M. tuberculosis ATCC 35825	Drugs used in combination (µM)		*Log₁₀ cfu drop vs. D0 Control*		Log_{10} cfu drop vs. D3 Control	Significance	Log_{10} cfu drop vs. D7 Control	Significance
	RIF	Drils-1398	Day 3	Day 7	Day 3		Day 7	
Combination 1	19.45	15	1.31	2.19	1.56	***	2.62	***
Combination 2	19.45	7.5	1.18	1.74	1.42	***	2.17	***
Combination 3	19.45	3.75	1.12	1.70	1.36	***	2.13	***
Combination 4	19.45	1.8	1.05	1.61	1.29	***	2.03	***
Combination 5	19.45	0.93	0.99	1.55	1.23	***	1.97	***
Drugs alone	19.45	-	0.98	1.54	1.23	***	1.96	***
	-	15	0.12	0.35	0.36	**	0.77	**
Cell control			-0.25	-0.42				

Fig. S9: Efficacy of DRILS-1398 in combination with Rifampicin against intracellular MDR *M. tuberculosis* ATCC 35825 in activated THP-1 macrophages, related to Fig. 4. A detailed analysis of Log_{10} cfu reduction and its statistical significance for each combination dosage are shown.

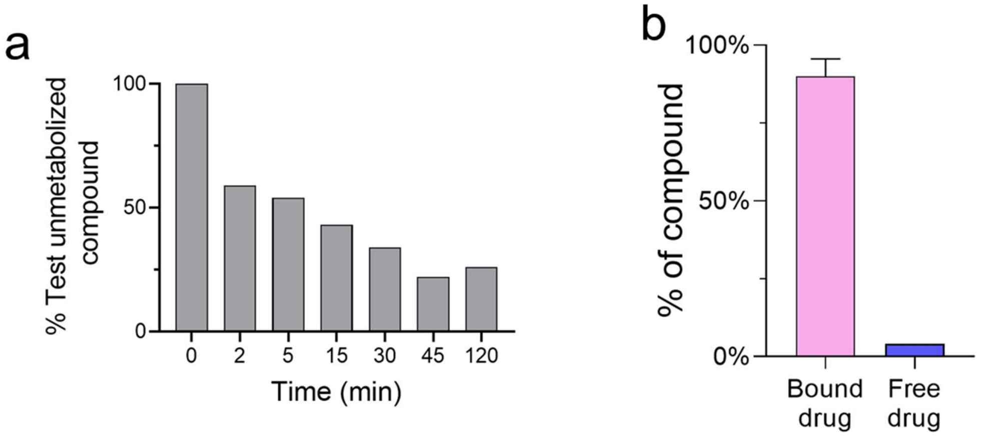

Fig. S10: Safety profile of DRILS-1398 (a) Mouse microsomal stability assay of DRILS-1398. (b) Mouse plasma protein assay of DRILS-1398.

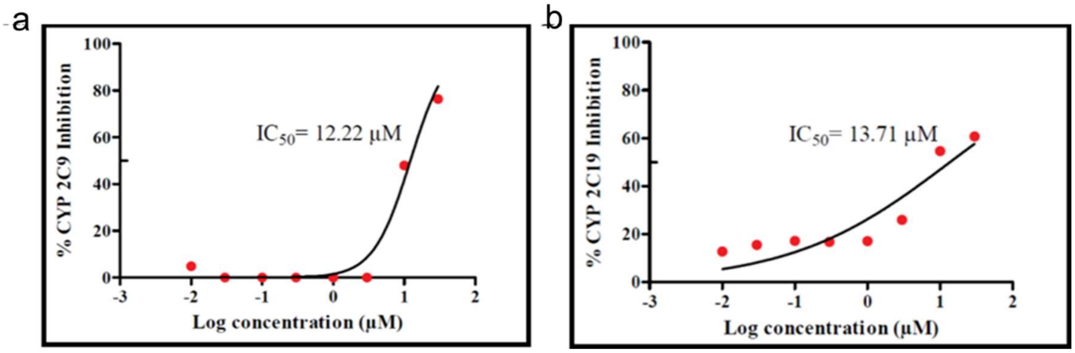

Fig. S11: ***In vitro*** **CYP enzyme inhibition study of DRILS-1398 by fluorescent substrate method in (a) CYP 2C9 and (b) CYP 2C19.** The percentage of CYP inhibition compared to the vehicle control was calculated. The mean CYP inhibition values from duplicate reactions were plotted against the log-transformed concentrations of DRILS-1398 to calculate the IC_{50}.

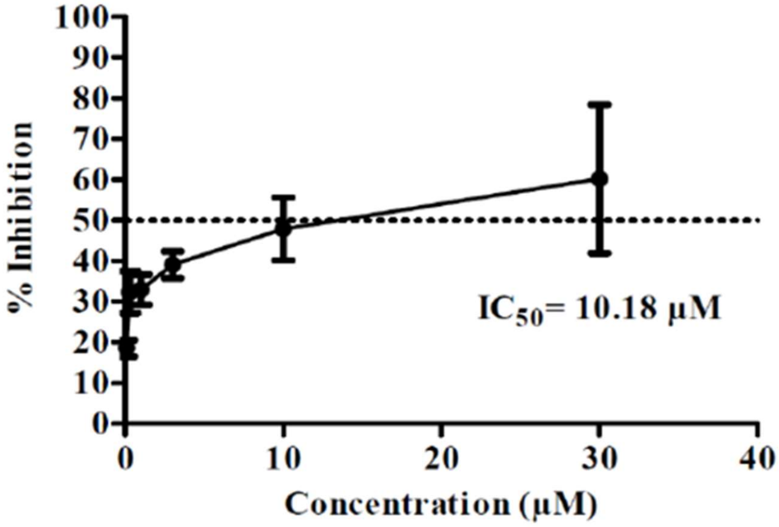

Fig. S12: *In vitro* evaluation of the effect of DRILS-1398 on hERG channel (IKr) over-expressed in human embryonic kidney cells. The Patch Control software was used to prepare and maintain the whole-cell configuration, and Patch Master software from HEKA was used to collect the electrophysiology data. The percentage of hERG inhibition was calculated and compared to that of vehicle control. The inhibition values from three trials were plotted against DRILS-1398 concentration to determine IC_{50} values.

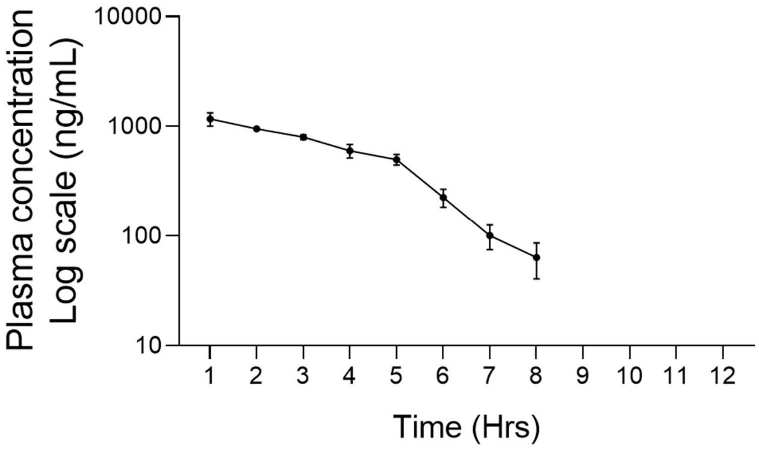

Fig. S13: Single dose IV (10 mg/kg) pharmacokinetics study of DRILS-1398 in male BALB/c Mice. Pharmacokinetics data was analyzed using Phoenix WinNonlin 8.1 software, and a non-compartmental model was selected for analysis. The mean and SD for the plasma concentrations were calculated using Microsoft Excel.

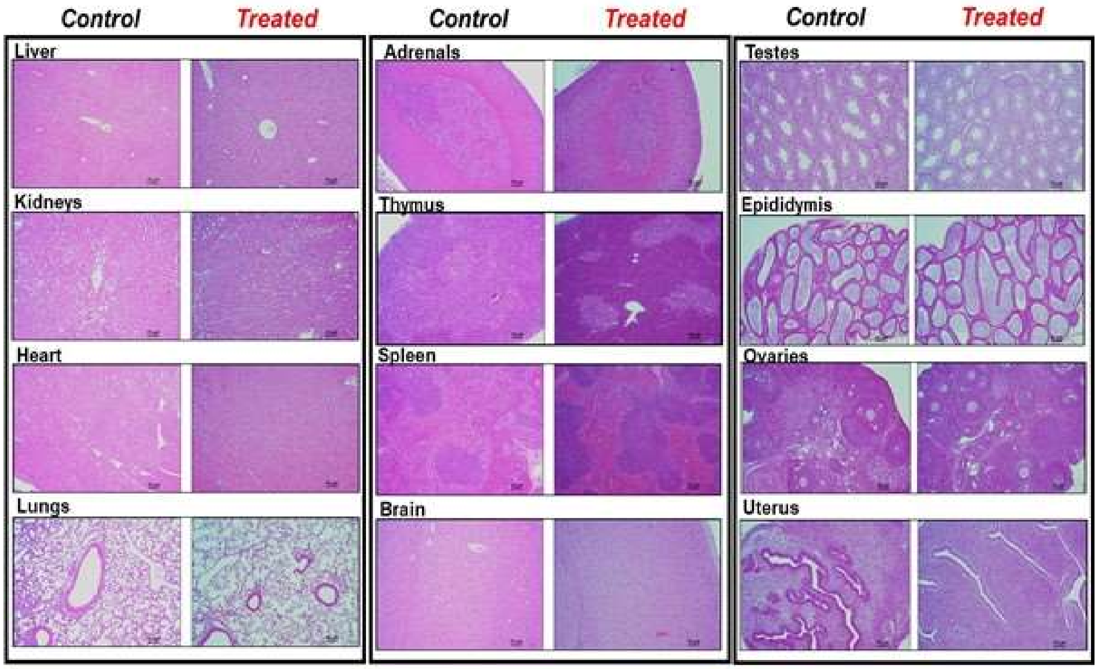

Fig. S14: Histopathological data of the 12 organs of control and DRILS-1398 (500 mg/kg body weight/day for seven consecutive days) treated BALB/c mice of both genders.

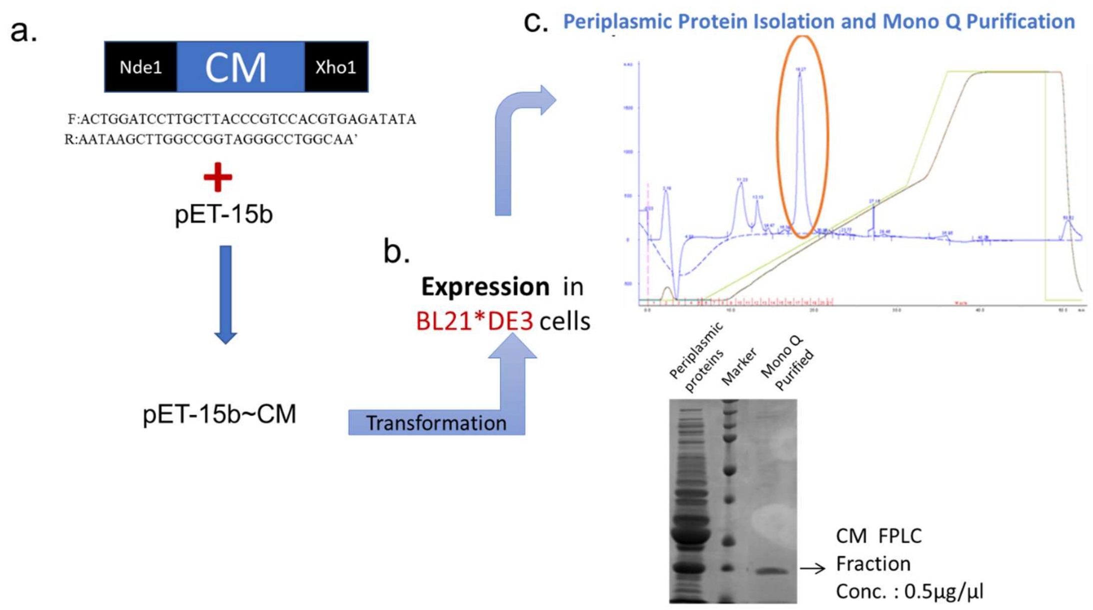

Fig. S15: Cloning, expression and purification of chorismate mutase enzyme (a) Cloning of chorismate mutase into pET-15b vector (b) expression of the cloned pET-15b-CM in BL21*DE3 cells and (c) Isolation and Mono Q purification of *M.tb*-CM enzyme.

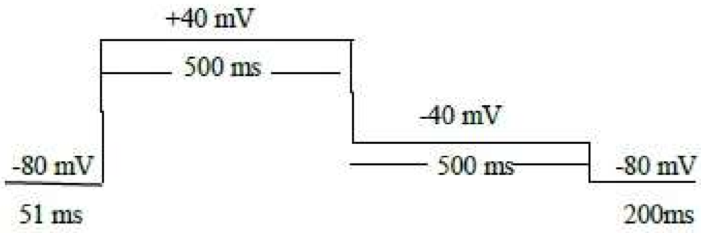

Protocol	Voltage (milli-volts)	Duration(milli-seconds)
Holding potential	-80	51
Depolarization	+40	500
Repolarization	-40	500
Holding potential	-80	200

Fig. S16: A schematic diagram of the Pulse protocol.

Table S1: ***In vitro*** **CYP enzyme inhibition study of DRILS-1398.**

Test/ Positive control items	Highest tested concentration (μM)	CYP 1A2	CYP 2C9	CYP 2C19	CYP 2D6	CYP 3A4
		IC50 (μM)				
**DRILS-1398 (Test item)**	**30**	**>30**	**12.22**	**13.71**	**>30**	**>30**
Furafylline (Positive control)	30	1.55	-	-	-	-
Sulfaphenazole (Positive control)	10	-	0.45	-	-	-
Tranylcypromine (Positive control)	50	-	-	0.94	-	-
Quinidine (Positive control)	1	-	-	-	0.002	-
Ketoconazole (Positive control)	1	-	-	-	-	0.05

Table S2: Mean plasma PK parameters of single-dose oral PK of DRILS-1398 at 50 mg/kg in mice, related to Fig. 5a.

Parameters	DRILS-1398
Route of administration	Oral
Dose (mg/kg b.w.)	**50**
Formulation vehicle	1% (v/v) Tween 80 + 99% (v/v) of 0.5% (w/v) Carboxymethylcellulose sodium salt in ultrapure water
C_{max} (ng/mL)	**1580.00**
T_{max} (hr)	4.00
AUC_{last} (hr*ng/mL)	**19189.66**
AUC_{inf} (hr*ng/mL)	21393.45
AUC_{extrap}(%)	10.30
$T_{1/2}$ (h)	6.95
MRT_{last} (h)	7.52

Table S3: Oral Pharmacokinetics Study of DRILS-1398 (F) in BALB/c Mice at 10 and 30 mg/kg, related to Fig. 5b.

Mean Plasma PK Parameters		
**Route of administration**	**Oral**	
Dose (mg/kg b.w.)	30.00	10.00
C_{max} (ng/mL)	4193.54	3705.38
T_{max} (hr)	6.00	4.00
AUC_{last} (hr*ng/mL)	66623.99	33831.54
AUC_{inf}(hr*ng/mL)	88761.16	34169.81
$AUC_{\% extrap}$(%)	24.94	0.99

$T_{1/2}$ (hr)	11.05	3.47
MRT $_{last}$ (hr)	9.52	6.19

Table S4: Histopathological analysis of the lungs of uninfected and *M.tb*-H_{37}Rv infected mice with and without drug treatment, related to Fig. 6. The scoring for the Inflammation, Granuloma, Lympho-Histiocytic collection, Necrosis was done. ZN staining was done to assess the presence of acid Fast bacilli (- : No Bacilli, + : bacilli present)

**Treatment**	**Inflammation**		**Granuloma**<br>**- : Absent**<br>**+ : Present**	**Lympho-Histiocytic collection**	**Necrosis**	**ZN stain (Acid Fast Bacilli)**<br>**- : Absent**<br>**+ : Present**
	**Mild**	**Moderate**				
Uninfected	✓		-	-	-	-
Infected		✓	+	+	-	+++++
Infected + DRILS-1398	✓		-	+	-	++
Infected + DRILS-1398 (F)	✓		-	+	-	++
Infected + ATT drug	✓		-	+	-	+
